# Application of non-invasive low strength pulsed electric field to EGCG treatment synergistically enhanced the inhibition effect on PANC-1 cells

**DOI:** 10.1371/journal.pone.0188885

**Published:** 2017-11-29

**Authors:** Chih-Hsiung Hsieh, Chueh-Hsuan Lu, Wei-Ting Chen, Bo-Lun Ma, Chih-Yu Chao

**Affiliations:** 1 Department of Physics, Lab for Medical Physics & Biomedical Engineering, National Taiwan University, Taipei, Taiwan; 2 Biomedical & Molecular Imaging Center, National Taiwan University College of Medicine, Taipei, Taiwan; 3 Institute of Applied Physics, National Taiwan University, Taipei, Taiwan; University of South Alabama Mitchell Cancer Institute, UNITED STATES

## Abstract

Traditional therapies for pancreatic cancer are usually expensive and likely to cause side effects, and most patients have the risk of recurrence and suffering pain. Here, we investigated combination treatment of epigallocatechin-3-gallate (EGCG) and non-invasive low strength pulsed electric field (PEF) on the human pancreatic cell line PANC-1. Cells were cultured in various concentrations of EGCG and exposed to trains of PEF. The results showed that the low strength PEF alone or single treatment with low concentration of EGCG did not obviously affect the cell proliferation and migration in PANC-1. However, the EGCG-induced inhibitions of cell viability and migration ability in PANC-1 were dramatically enhanced by the further exposure of low strength PEF (60 V/cm). In particular, the same combination treatment caused less inhibition of cell viability in non-malignant HEK293 cells. We also found the combination treatment significantly decreased the ratio of Bcl-2/Bax protein and increased caspase activity in PANC-1 cells, resulting in the promotion of apoptotic responses, evidenced by chromatin condensation. The findings of the present study reveal the synergistic reactions in the combination treatment may severely disturb mitochondria, enhance the intrinsic pathway transduction, and effectively induce apoptosis; moreover, the migration and invasion of PANC-1 cancer cells were also significantly suppressed. Since normal cells are less sensitive to this combination treatment, and the non-invasive PEF could be modified to focus on a specific location, this treatment may serve as a promising method for anti-cancer therapy.

## Introduction

Pancreatic cancer is an aggressive malignant tumor and the fourth leading cause of cancer-related deaths in men and women [1]. Despite therapeutic advances, it is difficult to make an early diagnosis, and the five-year survival rate is only about 5% of patients [2–4]. The high mortality of pancreatic cancer could be partly due to the drug resistance and invasive characteristics of cancer cells [5, 6]. Conventional medical and surgical treatments are usually ineffective for metastatic pancreatic cancer. Therefore, increasing drug sensitivity and inhibiting metastasis are two important strategies for the development of an efficient treatment for patients diagnosed with this dismal disease [6].

Currently, common treatments for pancreatic cancer are surgery, chemotherapy, and radiation therapy. However, these treatments often cause unpleasant side effects, and the patients still have a high risk of tumor recurrence [7, 8]. A new technology employing nanosecond high-voltage electroporation has been utilized as a novel treatment for local inhibition of cancer cells [9–11]. Many previous studies have reported this method could inhibit proliferation and induce apoptosis in various cancer cell lines in vitro [12–14]. Besides, in vivo studies have shown that nanosecond electroporation reduced the tumor size and inhibited secondary tumor growth [15, 16]. However, the treatment employing a serious of high field strength (> 1000 V/cm) pulses with ultrashort duration in nanoseconds induces not only apoptosis but also necrosis, which can result in undesirable inflammatory reactions [10, 14, 17]. In addition, a recent study has reported that high-voltage electroporation causes irreversible cell damage and tissue ablation [18]. On the contrary, low-voltage electroporation can increase the permeability of cell membranes and effectively induce cell apoptosis with less cell damage [19, 20], but its anti-cancer effect is not quite significant [17, 21]. Moreover, the electroporation by means of direct contact of the cells with electrodes [19, 22] may result in undesirable leakage current and could be dangerous for therapy [23, 24]. Recently, high intensity PEF exposure using indirect contact with electrodes was calculated and shown to induce biological effects [25–27]; nevertheless, the electric intensities employed in these studies (> 1000 V/cm) are too high and on the verge of dielectric breakdown, which is hazardous if electric current travels through the body [28]. Hence, we first propose that anti-cancer treatment with non-invasive low strength pulsed electric field (PEF) would be more suitable for patients.

In recent years, natural compounds with potent anti-cancer benefits have gained popularity, and it is believed these agents would cause fewer side effects and be more suitable for patients [29]. Epigallocatechin-3-gallate (EGCG), the most abundant catechin in green tea extracts, has antitumor activity against a broad spectrum of cancer [30, 31], such as human osteogenic sarcoma (HOS) cells [32], laryngeal squamous carcinoma cells [6], nasopharyngeal carcinoma cells [33], and pancreatic cancer cells [34]. In addition, EGCG exhibits powerful antioxidant properties and prevents inflammation-associated carcinogenesis [30]. However, these anti-cancer effects of EGCG were achieved at several hundred μM, which would also be cytotoxic for normal cells [35]. Besides, high concentrations of EGCG were reported to induce side effects, such as anxiolytic activity [36], hypoglycemic activity [37], and hypochromic anemia [38, 39]. Therefore, low concentration of EGCG with fewer side effects would be more applicable for anti-cancer treatment.

In this study, we investigate whether the inhibition effects of low concentration of EGCG on PANC-1 cells were enhanced by further application of non-invasive low strength PEF. It was observed that the inhibition efficiency of low concentration of EGCG on PANC-1 cells was drastically enhanced by further exposure of the cells to 60 V/cm PEF. In particular, no inhibition of cell viability was observed in the non-cancer HEK293 cells under the treatment with the same condition. This study provides the first demonstration of the application of non-invasive low strength PEF to low concentration EGCG treatment, and it shows remarkable anti-cancer properties. Our experiments revealed this combination treatment induced synergistic reactions and enhanced the inhibition effect on PANC-1 cells, and these results might shed light on the anti-cancer effects of combination treatment with natural agents and physical stimulation.

## Materials and methods

### Cell culture

The human pancreatic cancer cell line PANC-1 and the human embryonic kidney 293 (HEK293) were obtained from Bioresource Collection and Research Center (BCRC) (Hsinchu, Taiwan), and each STR-PCR profile had been performed for cell line authentication at BCRC. Both cell lines were maintained in Dulbecco’s modified Eagle’s medium (DMEM) (HyClone, South Logan, UT, USA) supplemented with 10% fetal bovine serum (FBS) (HyClone), 100 unit/ml penicillin (Gibco Life Technologies, Grand Island, NY, USA), and 100 mg/ml streptomycin (Gibco Life Technologies) at 37°C in humidified air with 5% CO_2_. Exponentially growing cells were harvested with 0.05% trypsin–0.5 mM EDTA solution (Gibco Life Technologies) and prepared for in vitro experiments.

### EGCG treatments

Epigallocatechin-3-gallate (EGCG) was purchased from Sigma-Aldrich (St. Louis, MO, USA) and dissolved in distilled water as a stock solution. PANC-1 and HEK293 cells were seeded into 35-mm-diameter culture dishes (1 × 10^5^ cells/dish) and then treated with various concentrations of EGCG (0, 10, 20, 30, 40, and 50 μM). After the cell viability assay, only 10 and 20 μM of EGCG were used in the following experiments.

### Experimental setup for exposure of the cell to PEF

**Fig 1:**
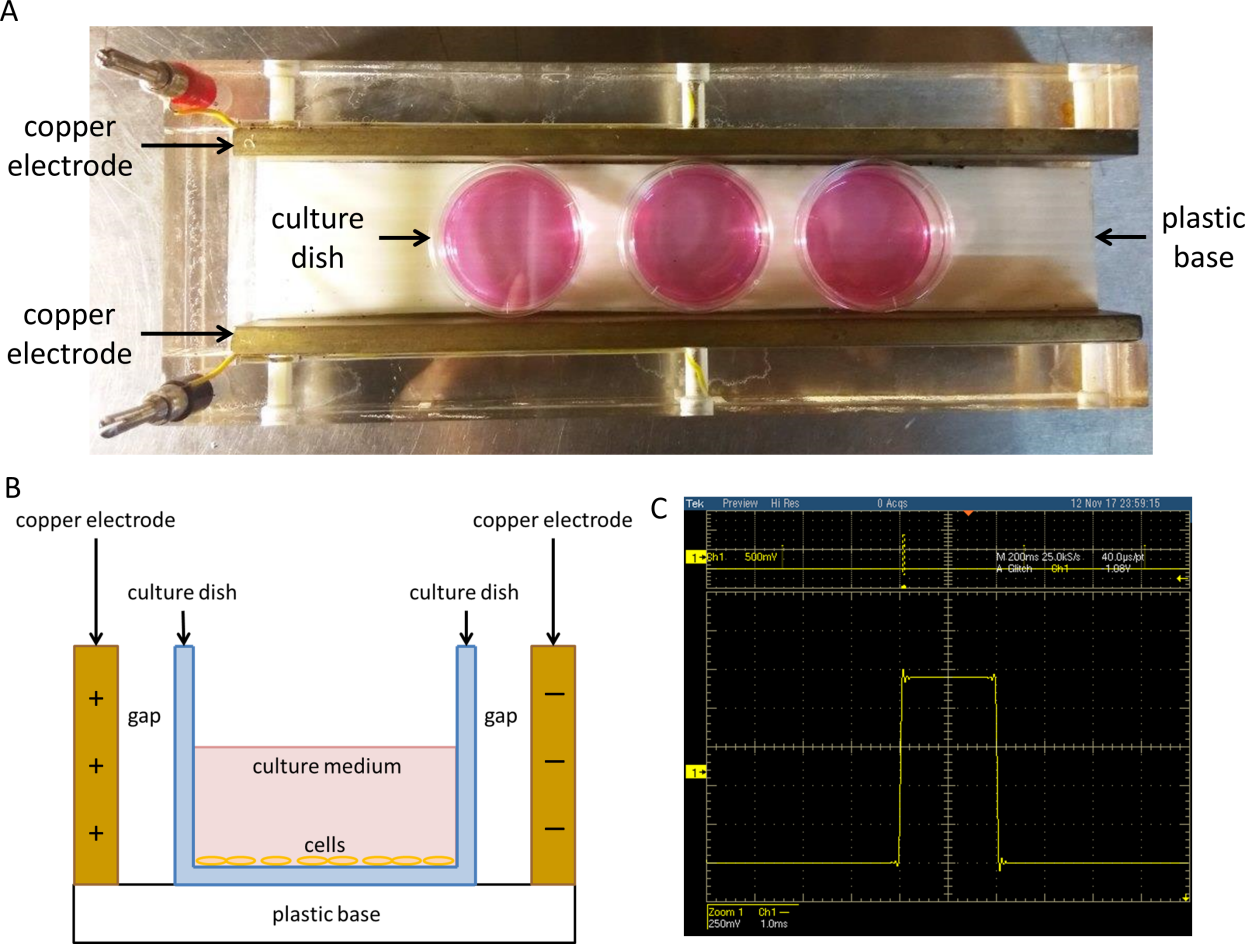
The device for exposure of the cells to PEF. (A) The cells treated with or without EGCG were placed between two thin copper flat and parallel electrodes (18 cm long at 3.5 cm distance). Electric fields of 15, 30, 60, and 90 V/cm were applied. (B) Separated by an insulating container and an air gap, the cells did not directly contact with the electrodes. (C) The waveform of 60 V/cm PEF was recorded using an oscilloscope.

The cells seeded in 35-mm-diameter dishes were treated with or without EGCG. The dishes were then placed between two copper flat and parallel electrodes (18 cm long at 3.5 cm distance) (Fig 1A). Since there were an insulating container and an air gap separating the electrodes from the cells, the two electrodes did not directly contact with the cells (Fig 1B). Consecutive pulses with the utmost voltage peak value 0.52, 1.2, 2.8, and 7.6 V, pulse width 2 ms, and the repetition frequency 2 Hz were produced by a function generator (Agilent 33220A; Agilent Technology, Palo Alto, CA, USA). The voltage of the output from the generator was increased through a power amplifier (Trek PZD700; Trek Inc., Medina, NY, USA). As a result, the voltage pulses were adjusted and electric fields of 15, 30, 60, and 90 V/cm were applied to the electrodes. Cells with different treatments were kept at 37°C in a humidified atmosphere of 5% CO_2_. The power amplifier supplies a buffered voltage (1/200th, ±0.1% of full scale) for monitoring the output waveform. Fig 1C showed the waveform of 60 V/cm PEF, measured by an oscilloscope (TDS5034B; Tektronix, Beaverton, Oregon, USA).

### Cell viability assay

3-(4,5-dimethylthiazol-2-yl)-2,5-diphenyltetrazolium bromide (MTT) (Sigma-Aldrich) was dissolved in distilled water as a 5 mg/ml stock solution. Cell viability and cell growth were measured using the MTT assay. After cells were treated with EGCG, PEF, or EGCG plus PEF for 24 h (cell viability) or 72 h (cell growth) at 37°C with 5% CO_2_, the medium was replaced with fresh one containing 0.5% mg/ml MTT followed by a 4 h incubation at 37°C. The supernatant was removed, and 2 ml of DMSO was added to dissolve the formazan crystals. The absorbance was read at 570 nm using Multiskan GO microplate spectrophotometer (Thermo Scientific, NH, USA).

### Immunostaining

Cells were grown on sterile glass coverslips, treated with different conditions for 24 h, fixed in 4% paraformaldehyde (Sigma-Aldrich) for 15 min, and then permeabilized with 0.1% Triton X-100 (Bioshop Canada Inc, Burlington, Ontario, Canada) in PBS for 15 min at room temperature. After washing twice with PBS, the cells were blocked with 1% bovine serum albumin (BSA) (Bioshop Canada Inc) in PBS for 1 h. The cells were then incubated overnight with primary anti-caspase 3 (1:1000 dilution, Cell Signaling Technology, Danvers, MA, USA) at 4°C. After three rinses with PBS, Alexa Fluor 647 anti-rabbit secondary antibody (1:1000 dilution, Abcam, Cambridge, MA, USA) diluted in blocking buffer was applied to the cells for 1 h. Finally, the coverslips were mounted in 10 μl of Glycerol Mounting Medium contained DAPI (Abcam). Localization of cleaved caspase-3 and morphological changes of the nuclei were observed using a Zeiss Axio Imager A1 fluorescence microscope.

### Apoptosis analyses by flow cytometry

After treatments for 24 h, PANC-1 cells were collected and washed twice with PBS. The cells were then transferred to 100 μl of binding buffer and incubated with an Annexin V-FITC/Propidium Iodide (BD Biosciences, San Jose, CA, USA) staining solution for 15 min at room temperature in the dark, and then added with 400 μl of binding buffer into each tube for final flow cytometry measurement. Statistical analysis of fluorescence was recorded by FACSCanto™ II system (BD Biosciences).

### Western blotting analysis

After treatments for 24 h, PANC-1 cells were washed with PBS and lysed in lysis buffer (50 mM Tris-HCl, pH 7.4, 0.15 M NaCl, 0.25% deoxycholic acid, 1% NP-40, 1% Triton X-100, 0.1% SDS, 1 mM EDTA) containing fresh protease and phosphatase inhibitor cocktail (Millipore, Billerica, MA, USA) over ice for 30 min. After centrifugation and supernatant collection, the protein concentrations were quantitated by BSA method. Equal amounts of lysate were separated by 12.5% SDS-PAGE and transferred to polyvinylidene difluoride (PVDF) membranes (Millipore). The blots were blocked with 50 g/L nonfat milk in TBST washing buffer (20 mM Tris, 150 mM NaCl, and 0.1% Tween 20) for 1 h and incubated overnight with primary antibodies at 4°C, followed by incubation with horseradish peroxidase-coupled secondary antibodies for 1 h. In this study, primary antibodies were purchased from the following: Anti-β-actin (Gentex, Irvine, CA, USA); anti-Bax (Santa Cruz Biotechnology, Santa Cruz, CA, USA); anti-Bcl-2, anti-cleaved caspase-3, and anti-cleaved caspase-9 (Cell Signaling Technology). The secondary antibodies were purchased from Jackson ImmunoResearch Laboratories (West Grove, PA, USA). All the antibodies were diluted at the optimal concentration according to the manufacturer's instructions. Following, the detected proteins were visualized using WesternBright ECL Western blotting detection kit (Advansta Inc., Menlo Park, CA, USA). β-actin was used as the internal control to correct relative levels of each protein loading.

### Mitochondrial membrane potential detection

The dissipation of mitochondrial membrane potential (MMP) was assessed using 3,3-dihexyloxacarbocyanine iodide (DiOC_6_) (Enzo Life Sciences International Inc., NY, USA) [40]. After treatment for 24 h, PANC-1 cells were collected and washed twice with PBS. The cells were then incubated with PBS containing 20 nM DiOC_6_ at 37°C for 30 min in the dark. After that, the stained cells were washed with PBS and resuspended in 500 μl PBS for final flow cytometry measurement. Statistical analysis of fluorescence was recorded by FACSCanto™ II system (BD Biosciences).

### Wound healing assay

PANC-1 cells were seeded and grown to full confluence in 35-mm-diameter dishes. Wounds were created by scratching straight lines in the monolayer cells with a 10 μl pipette tip, and the cells were then gently rinsed with PBS to remove non-adherent cells and debris. After PBS was discarded and replaced with fresh medium, the cells were applied to different treatment conditions. Each wound was observed and photographed by the Zyla 5.5 microscope after treatment for 24 h. The distances between wound edges were measured and analyzed using Image J, and the results were presented as the average percentage of wound closure compared with time zero.

### Statistical analysis

Each experiment was repeated three times, and statistical analysis was performed using SigmaPlot version 12.5 for Windows (Systat Software, Inc., San Jose, CA, USA). The one-way analysis of variance (ANOVA) was employed for comparing the means between each group, and a value of P<0.05 was considered statistically significant. In the figures, * is used for P < 0.05, ** for P<0.01, and *** for P < 0.001.

### Synergy quotient calculation for synergism

The synergism quotient (SQ) was evaluated by deducting baseline values from all treatments and then dividing the net effect of the combination [A + B] by the sum of individual effects [A] + [B]. SQ greater than 1.0 reveals a synergistic effect.

## Results

### Application of low strength PEF promotes the inhibition effect of EGCG on PANC-1 cells

**Fig 2:**
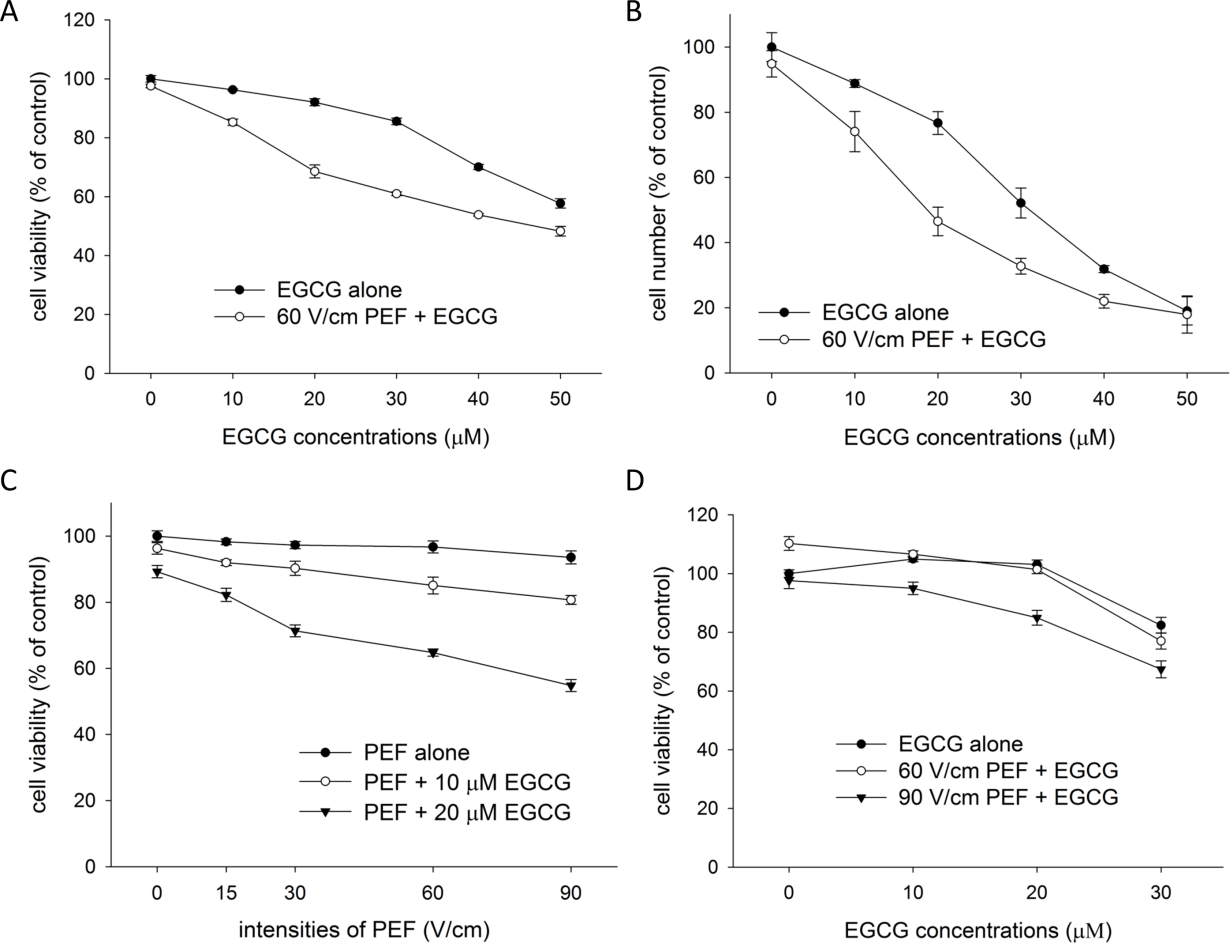
Effects of PEF on the EGCG-induced growth inhibition in PANC-1 and HEK293 cells. The cell viability (24 h) and the cell number (72 h) were determined using MTT assay. PANC-1 cells were treated with various concentrations of EGCG alone or combined with consecutive PEF (60 V/cm in amplitude, 2 ms in duration, and 2 Hz in frequency) for (A) 24 h or (B) 72 h. EGCG (0–50 μM) alone concentration-dependently inhibited PANC-1 cells, whereas co-treatment with EGCG and the PEF together enhanced the inhibition. (C) PANC-1 cells were treated with EGCG (0–20 μM) alone or combined with various strengths of PEF for 24 h. (D) HEK293 cells were treated EGCG (0–30 μM) alone or combined with PEF (60 or 90 V/cm) for 24 h.

We first investigated whether low strength PEF could enhance the ability of EGCG to inhibit cell growth. We employed the PEF with parameters of 60 V/cm in amplitude, 2 ms in duration, and 2 Hz in frequency in our experiments. As shown in Fig 2A and 2B, PANC-1 cells were treated with EGCG (from 0 μM to 50 μM) alone or combined with consecutive PEF for 24 and 72 h. After treatments, the growth inhibition effects of single and combined treatments were evaluated using MTT assay. The results show that EGCG alone reduced cell viability (24 h) and cell number (72 h) in a dose-dependent manner, and co-treatment with 60 V/cm PEF and EGCG further enhanced the reduction in cell viability (24 h) and cell number (72 h). As shown in Table 1, the synergy quotient (SQ) calculation of cell viability (24 h) indicated that a synergistic effect was found when 60 V/cm PEF was applied to the treatment of EGCG at concentrations from 10 to 50 μM. The largest two values of synergy quotient were obtained in the combination treatment using 10 and 20 μM of EGCG, and this result revealed the synergistic effects of combination treatments using the lower concentrations of EGCG were better than those using the higher concentrations. Besides, after a continuous co-treatment of the PEF and EGCG (10 or 20 μM) for 72 h, the number of viable cells was further reduced when compared with the viability of cells treated with the same treatment for 24 h, suggesting this gentle combination treatment would be more beneficial as a long-term remedy. Next, as shown in Fig 2C, various intensities PEF (15 to 90 V/cm) were applied to combination treatment to better understand the effect of electric field strength on the EGCG-induced growth inhibition in PANC-1 cells. We found the cell viability was still high while cells were single treated with 90 V/cm PEF. However, the inhibition effect of EGCG was synergistically enhanced even by the application of 15 V/cm PEF. Besides, it was observed that the inhibition effect of the combination treatment was synergistically enhanced not only in a dose-dependent manner by EGCG, but also in an intensity-dependent manner by PEF. Following, we employed similar experiments using non-malignant embryonic kidney HEK293 cells to understand whether the combination treatment had high specificity to PANC-1 cells. Interestingly, Fig 2D shows HEK293 cells were less sensitive to the combination treatment using EGCG (10 or 20 μM) and 60 V/cm PEF. Nevertheless, when HEK293 cells were co-treated with 90 V/cm PEF and EGCG (10 to 30 μM), the cell viability was notably reduced. These findings proposed that co-treatment with EGCG (10 or 20 μM) and 60 V/cm PEF would be helpful to inhibit the growth of pancreatic cancer cells with less adverse effect on the body. Based on these results, we used low concentrations (10 and 20 μM) of EGCG and 60 V/cm PEF for the following experiments.

**Table 1:** Synergy quotient of PEF on the EGCG-induced growth inhibition.

	Cell Viability (% control)	Inhibition Rate	SQ Value
**PEF**	97.57 ± 0.59	2.43	
**10 μM EGCG**	96.26 ± 0.30	3.74	
**PEF + 10 μM EGCG**	85.28 ± 1.08	14.72	2.39
**20 μM EGCG**	92.07 ± 1.18	7.93	
**PEF + 20 μM EGCG**	68.56 ± 2.19	31.44	3.03
**30 μM EGCG**	85.57 ± 1.12	14.43	
**PEF + 30 μM EGCG**	60.97 ± 0.66	39.03	2.31
**40 μM EGCG**	70.07 ± 0.87	29.93	
**PEF + 40 μM EGCG**	53.85 ± 0.44	46.15	1.43
**50 μM EGCG**	57.71 ± 1.58	42.29	
**PEF + 50 μM EGCG**	48.24 ± 1.64	51.76	1.16

### Enhanced apoptotic effect of EGCG on PANC-1 cells in the presence of low strength PEF

**Fig 3:**
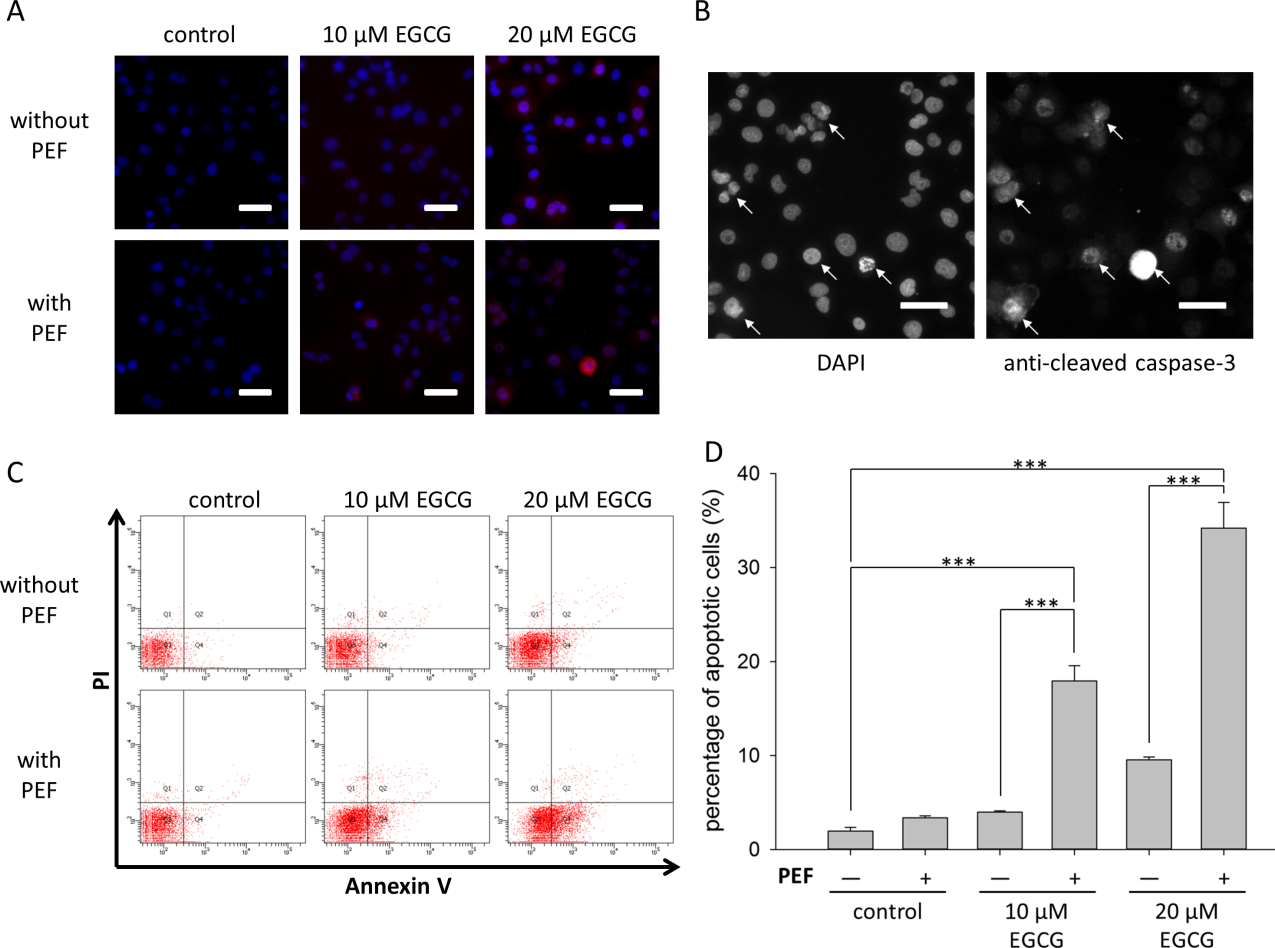
Effects of PEF on EGCG-induced apoptosis in PANC-1 cells. PANC-1 cells were treated for 24 h with EGCG (10 or 20 μM) alone or combined with 60 V/cm PEF. (A) The nuclei morphology was analyzed using DAPI staining (blue), and the apoptotic cells were revealed by cleaved caspase-3 labeling (red). Scale bar = 10 μm. (B) The double staining of cells co-treated with 20 μM EGCG and 60 V/cm PEF was presented as two separate enlarged images. The left and right images represented the DAPI-stained nuclei and the signals of cleaved caspase-3, respectively. It was observed that cells showing nuclear condensation (arrowheads) were also labeled with anti-cleaved caspase-3 (arrowheads). Scale bar = 10 μm. (C) Double staining Annexin V/PI and flow cytometry were employed to analyze the apoptotic effect of the combination treatment. (D) Apoptotic cells were considered as the sum of early and late apoptotic cells. The percentage of apoptotic cells were presented as the mean ± S.D. of triplicate experiments. A significant increase of apoptotic cells was identified in the cells co-treated with 10 μM EGCG and 60 V/cm PEF. Similarly, co-treatment with the PEF and 20 μM EGCG significantly increased the apoptotic cell number of PANC-1. The “+” means that the PEF was involved in the treatment, and the “-” means the PEF was not involved. (***, p<0.001 compared to corresponding control).

We further investigated the activity of caspase-3 and the change of nuclear morphology to examine whether the viability inhibition of this combination treatment was through apoptosis. As shown in Fig 3A, when cells were exposed to 60 V/cm PEF alone or single treated with EGCG (10 or 20 μM), the nucleus shape of PANC-1 cells was intact with homogenous fluorescence, and little activated caspase-3 expression was observed. In contrast, as shown in Fig 3B, when PANC-1 cells were co-treated with 20 μM EGCG and 60 V/cm PEF, chromatin condensation (left) and cells labeled with anti-cleaved caspase 3 (right) could be observed obviously. To better understand and quantify the apoptotic effect of combination treatment, double staining Annexin V/PI flow cytometry was employed. Annexin-V-positive and PI-negative cells are considered as early apoptotic, and the double positive cells are classified as late apoptotic or necrotic. Here, apoptotic cells were considered as the sum of early and late apoptotic cells. As illustrated in Fig 3C and 3D, co-treatment with EGCG and PEF led to a significantly higher level of Annexin V staining compared with the single treatment of EGCG. Notably, the combination treatment using 10 μM EGCG resulted in a higher percentage of apoptotic cells than the single treatment using 20 μM EGCG. Collectively, these results suggest that the growth inhibition effect of the combination treatment is closely related to apoptosis activities, and combination treatment using the lower concentration of EGCG could induce more apoptotic cells than single treatment using the higher concentration of EGCG.

### Combination treatment alters the protein levels of Bcl-2 and Bax

**Fig 4:**
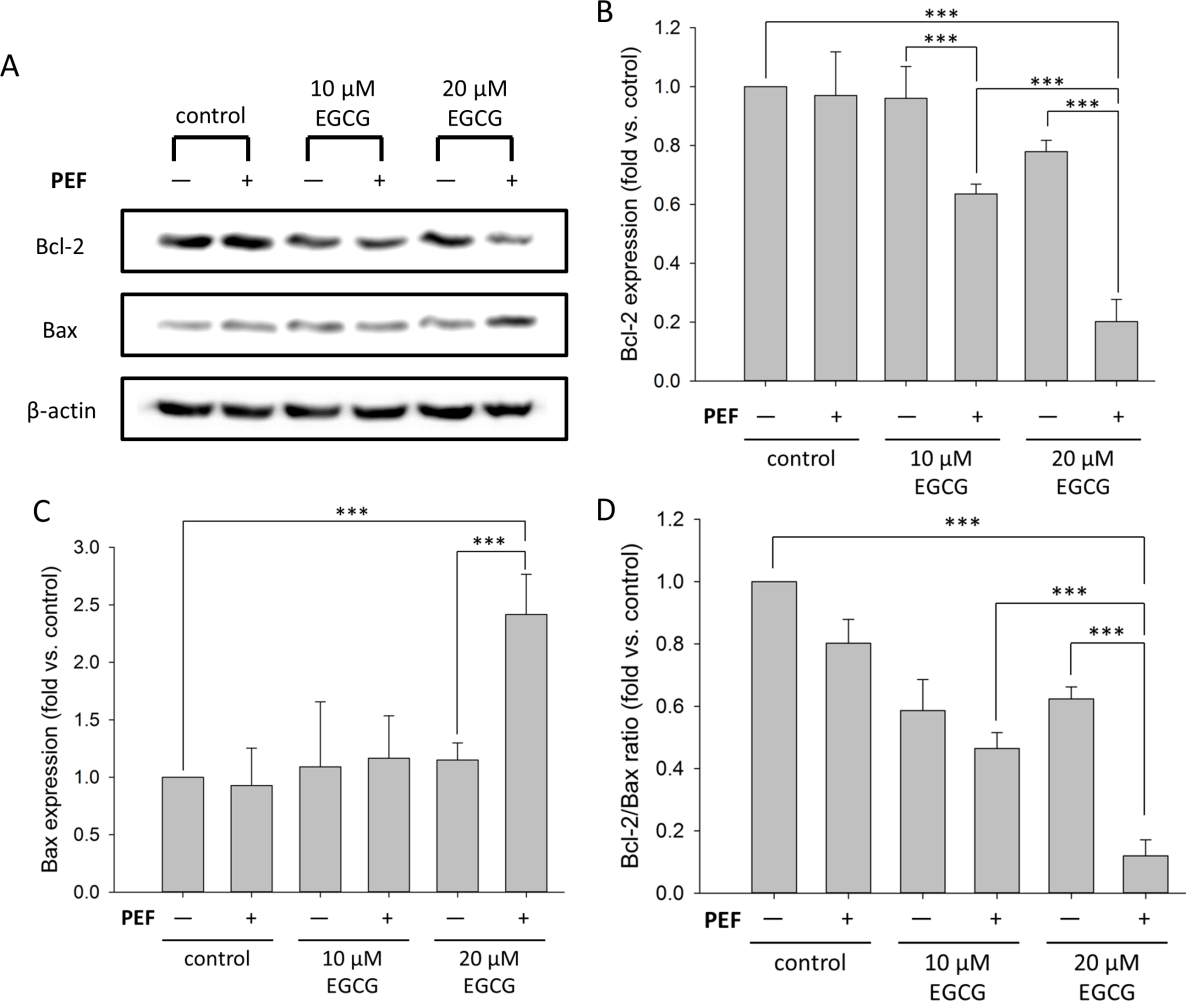
Effect of co-treatment with EGCG and PEF on the protein levels of Bcl-2 and Bax. PANC-1 cells were treated for 24 h with EGCG (10 or 20 μM) alone or combined with 60 V/cm PEF. (A) Protein expression levels of Bcl-2 and Bax were measured using Western blot analysis. Each relative expression level was normalized against β-actin and compared with control. The cells co-treated with 20 μM EGCG and 60 V/cm PEF showed significant decrease of Bcl-2 and increase of Bax. (B) Bcl-2 band intensities were quantified. When PANC-1 cells were co-treated with EGCG and PEF, Bcl-2 protein levels were significantly decreased. (C) Bax band intensities were quantified. The Bax protein level was significantly increased only when PANC-1 cells were co-treated with 20 μM EGCG and PEF. (D) The Bcl-2/Bax ratio was determined, and this result revealed that the combination treatment of low concentration EGCG and low intensity PEF promoted the down-regulation of Bcl-2/Bax ratio. The “+” means that the PEF was involved in the treatment, and the “-” means the PEF was not involved. (***, p<0.001 compared to corresponding control).

Since Bcl-2 family proteins play central roles in cell death regulation, we next studied the changes in the expressions of Bcl-2 and Bax. As shown in Fig 4A, the Western blot analysis revealed a compelling decrease in Bcl-2 protein and an increase in Bax protein when PANC-1 cells were co-treated with 60 V/cm PEF and 20 μM EGCG. Fig 4B and 4C show the quantified band intensities of Bcl-2 and Bax, respectively, and each expression value was normalized to β-actin and compared with control. The results revealed that the Bcl-2 expression levels of single treatment with EGCG (10 or 20 μM) were further reduced by the application of 60 V/cm PEF (Fig 4B). In addition, the Bax expression level was increased significantly when PANC-1 cells were co-treated with 60 V/cm PEF and 20 μM EGCG (Fig 4C). As a result, the Bcl-2/Bax ratio remarkably decreased when PANC-1 cells were co-treated with 60 V/cm PEF and 20 μM EGCG (Fig 4D). Therefore, it could be suggested that combination treatment with PEF and EGCG promoted the down-regulation of Bcl-2/Bax ratio and consequently enhanced the inhibition effects of EGCG.

### Dissipation of mitochondrial membrane potential caused by combination treatment

**Fig 5:**
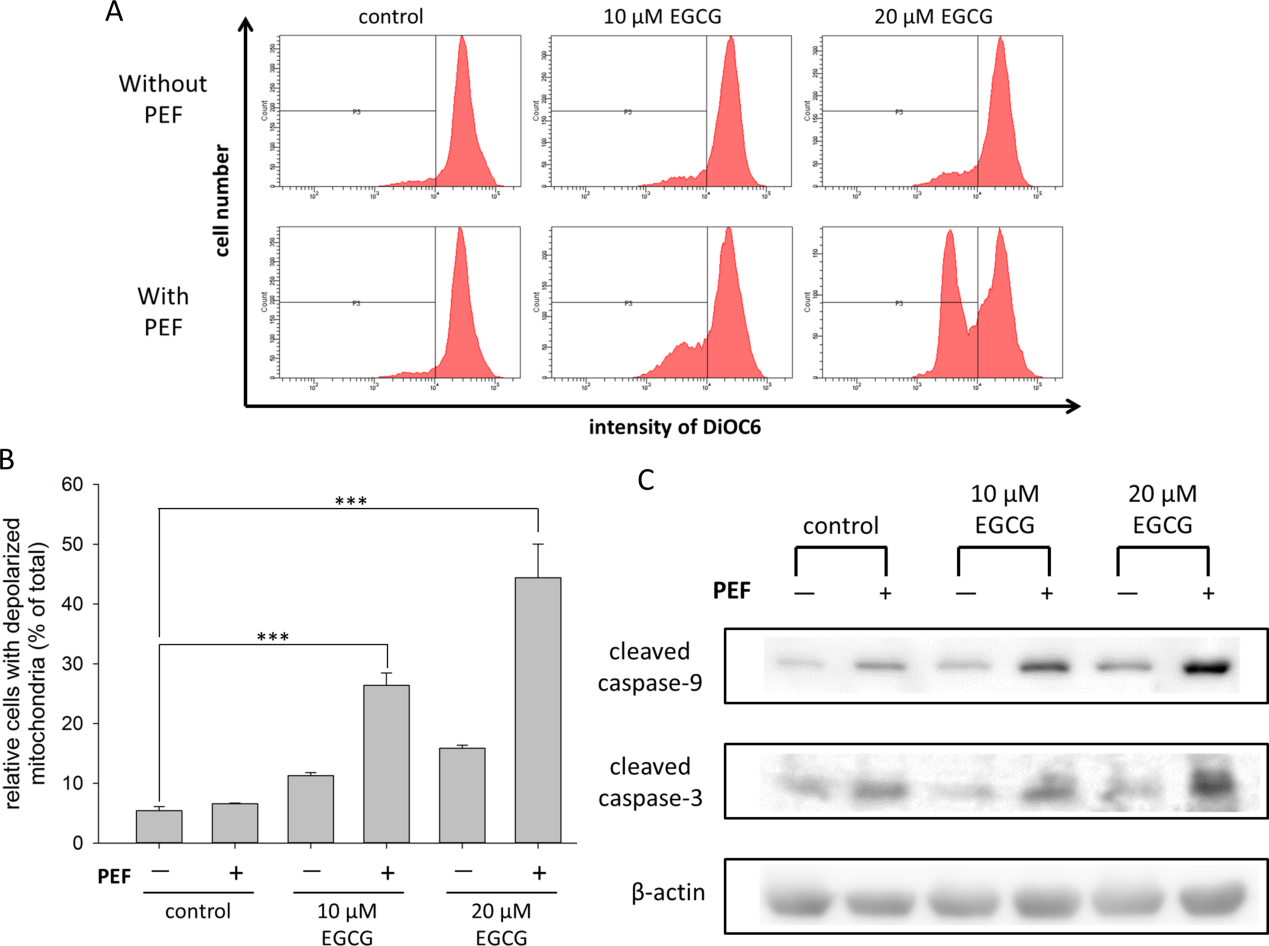
Effects of co-treatment with EGCG and PEF on mitochondrial-mediated apoptotic pathway. (A) The dissipation of mitochondrial membrane potential (MMP) was determined using DiOC_6_ staining. DiOC_6_ is a green-fluorescent and positively charged molecule that can accumulate in the polarized mitochondria, and the reduction of its fluorescence intensity indicated the depolarization of mitochondria. Our results showed the PEF could enhance the EGCG-induced MMP dissipation. (B) The percentage of cells with depolarized mitochondria was significantly increased when the PEF was applied to EGCG treatment. (C) Cleaved caspase-9 and -3 were detected by Western blot. β-actin was used as a loading control. This result showed that the EGCG-induced caspase activities were enhanced when cells were further exposed to the PEF. The “+” means that the PEF was involved in the treatment, and the “-” means the PEF was not involved.

We checked whether the mitochondrial function was affected by the combination treatment, so the DiOC_6_ fluorescence was used and measured to analyze the dissipation of mitochondrial membrane potential (MMP) [40]. As shown in Fig 5A and 5B, there was no significant difference between the control group and the groups single treated with 60 V/cm PEF or EGCG (10 and 20 μM). In contrast, cells co-treated with EGCG (10 or 20 μM) and the PEF showed significant reductions of DiOC_6_ fluorescence intensity. The percentages of cells with depolarized mitochondria increased from 11.3% to 26.4% and 15.9% to 44.4% after applying the PEF to 10 and 20 μM EGCG, respectively. This result indicated that the application of PEF to EGCG treatment could synergistically enhance the dissipation of MMP.

### Promotion of EGCG-induced caspase activities by the application of PEF

It is known that caspase activation plays an important role in the signal transduction of apoptosis, so we investigated the expression levels of cleaved caspase-9 and -3 in PANC-1 cells co-treated with 60 V/cm PEF and EGCG (10 μM or 20 μM). As shown in Fig 5C, the level of cleaved caspase-9 was increased in the cells treated with EGCG, and the increment became significant when EGCG-treated cells were further exposed to 60 V/cm PEF. This result suggested that the low strength PEF could affect mitochondrial function and trigger caspase-9 activity. Similar expression levels were obtained in the cleaved caspase-3 observation. The result indicated that the combination treatment with PEF and EGCG synergistically enhanced the caspase-dependent pathway. Therefore, our data revealed that the PEF disturbed mitochondrial polarization and enhanced the EGCG-induced apoptotic signal transduction.

### Combination treatment suppresses PANC-1 cells migration

**Fig 6:**
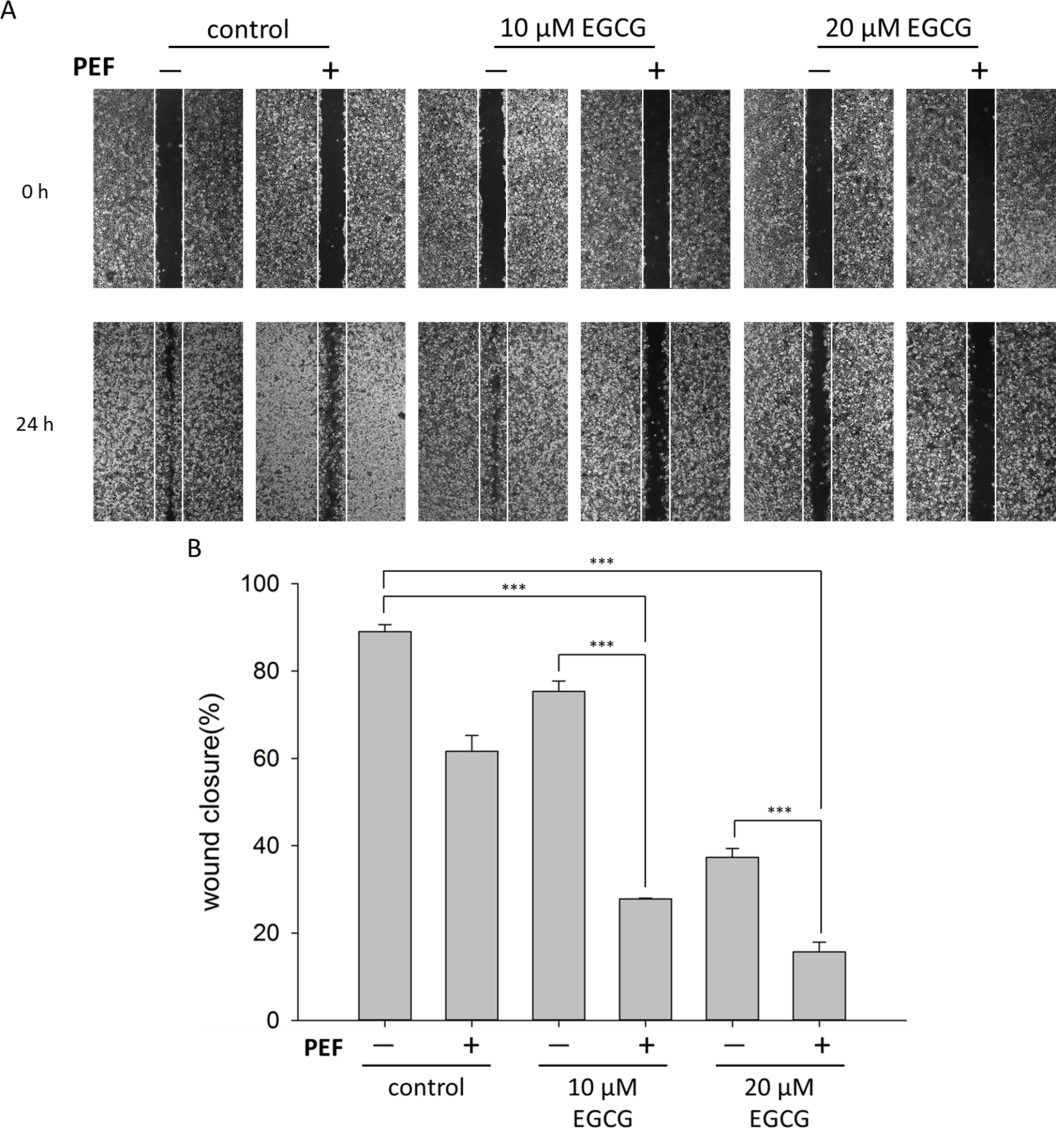
Effects of the combination treatment on the inhibition of PANC-1 migration. Effects of combination treatment on the migratory potential of PANC-1 cells were analyzed using wound healing assay. After scratch gap was created, PANC-1 cells were treated for 24 h with EGCG (10 μM or 20 μM) alone or combined with 60 V/cm PEF. (A) Representative pictures showed the scratch (wound) at t  =  0 h and 24 h. (B) The areas of cell-free gaps were measured and quantified using ImageJ. Wound closure rate was determined as the percentage of area closed after 24 h from the initial wound area. These results revealed combination treatment with EGCG and PEF significantly reduced the migration ability of PANC-1 cells. The “+” means that the PEF was involved in the treatment, and the “-” means the PEF was not involved. (***, p<0.001 compared to corresponding control).

Abnormally enhanced cell migration and invasion capabilities are associated with tumor metastasis, and pancreatic cancer is characteristic of early metastasis and high mortality. In our work, the classic wound healing assay was performed to examine the effect of the combination treatment on PANC-1 cells migration. As shown in Fig 6A and 6B, we found that PANC-1 scrambled control cells tended to reform a complete monolayer within 24 h, and cells single treated with PEF exhibited reduced migratory capability. When cells were co-treated with 60 V/cm PEF and 10 μM EGCG, the migration ability of aggressive pancreatic cancer cells was significantly suppressed. In addition, it is worth noting that there was a remarkable deviation of migration rate between the 10 μM EGCG group and the 10 μM EGCG plus PEF group (Fig 6B). The migration ability of cells treated with 10 μM EGCG was powerfully suppressed after further application of PEF. Similarly, co-treatment with the PEF and 20 μM EGCG significantly suppressed the migration ability of PANC-1 cells. Overall, these results revealed that PEF could attenuate the mobility of PANC-1 cells and significantly decrease the migration rate when it was applied to the EGCG treatment.

## Discussion

The novelty of this study is that the continuous exposure to non-invasive low strength PEF was applied to the cancer treatment with EGCG. We adopted the PEF with moderate parameters and found synergistic activities in the inhibitions of cell viability and migration when cells were co-treated with EGCG and PEF. In this study, it was observed that the combination treatment with 60 V/cm PEF and 20 μM EGCG significantly reduced the viability of PANC-1 cells, compared with the single treatment with 20 μM EGCG. Furthermore, the migration ability of PANC-1 cells was extremely suppressed when merely 10 μM EGCG was used in the combination treatment. We suggest that it could also show anti-cancer effects if the combination treatment employed a lower strength of PEF with different pulse repetition frequencies. In addition, the prominent SQ values were obtained at lower concentrations of EGCG, revealing an advantage of the combination treatment is lowering the concentration of EGCG for treatment, and it is much more gentle for therapy. Besides, it is worth to mention that co-treatment with 20 μM EGCG and 60 V/cm PEF caused little inhibition of cell viability in HEK293 cells; therefore, the combination treatment could be an effective therapy due to the distinction of superior sensitivity to PANC-1 cells.

In the experiments presented here, we quantified the percentage of apoptotic cells and relevant protein expressions of the combination treatment in PANC-1 cells. Our data showed that the combination treatment induced significantly more apoptotic cells than single treatments. Since normal cells were found to be less sensitive to the combination treatment than cancer cells, and the electric field could be modified to focus on a specific location, we suggest the non-invasive PEF would be a promising method for cancer therapy when it is used in combination with other natural agents or other physical stimulation.

Further, we were interested to understand the molecular mechanism underlying the synergistic effect of the combination treatment. Signaling pathways of programmed cell death can be subdivided into two major parts, the extrinsic (receptor-mediated) and the intrinsic (mitochondria-mediated) apoptotic pathways. The intrinsic pathway is initiated through the release of proapoptotic factors from mitochondria, and the permeabilization of the mitochondrial outer membrane is regulated by the proteins of the Bcl-2 family. Among the interactions between members of the Bcl-2 family, Bcl-2/Bax ratio serves as an important upstream checkpoint and functions as the mediator of mitochondrial apoptosis [41, 42]. According to our results, the protein expression of Bcl-2/Bax ratio was extremely decreased when cells were co-treated with 20 μM EGCG and 60 V/cm PEF. An analogous result was also obtained in the group co-treated with 10 μM EGCG and 60 V/cm PEF. It is noteworthy that the expression levels of Bcl-2 were significantly reduced when 60 V/cm PEF was applied to the EGCG (10 μM or 20 μM) treatment. This suggested that low strength PEF could strengthen the ability of EGCG to inhibit Bcl-2 expression. In addition, a previous study that used NMR spectroscopy demonstrated EGCG could bind to the hydrophobic pocket of Bcl-2 and inhibit its interaction with proapoptotic proteins, resulting in downregulation of the anti-apoptotic Bcl-2 [43]. Besides, it was reported that the application of PEF to soybean protein could increase its surface hydrophobicity [44]. Thus, we suggested the application of 60 V/cm PEF to EGCG treatment could enhance the hydrophobic binding between EGCG and Bcl-2 and therefore promote the activation of apoptotic signal transduction.

Furthermore, the high-frequency component of PEF was proposed to penetrate through cytomembrane and to affect intermembrane organelles, such as mitochondria and nucleus [19, 20, 45–47]. Recent calculations have also shown a little change of membrane voltage could be induced by non-invasive high strength PEF (> 1000 V/cm) [27], but the bio-effects induced by non-invasive low strength PEF alone or combined with natural agents remain unknown. Here, we suggested that the synergistic reactions triggered by combination treatment could intensify the disturbance of mitochondria and enhance the depolarization of MMP due to the application of PEF. In this study, we observed that the MMP of PANC-1 cells was significantly decreased, and the expression levels of cleaved caspase-9 and -3 were significantly increased when the cells were co-treated with EGCG (10 μM or 20 μM) and 60 V/cm PEF. Consequently, morphologic nuclear changes and chromatin condensation were observed as well by fluorescence staining in our experiments. These findings confirmed that the synergistic effects of the combination treatment disturbed PANC-1 mitochondria and initiated the mitochondria-mediated pathway, resulting in the promotion of apoptotic responses.

Since the inhibition rates of cell viability and migration were indirectly related to protein expression levels, the synergism of the combination treatment showed different effects on the viability and migration assay. When the 60 V/cm PEF was applied to 10 μM EGCG treatment, it showed significant inhibition in the cell migration but not in the cell viability. Moreover, both the migration and viability of PANC-1 cells were significantly suppressed by the combination treatment using 20 μM EGCG and 60 V/cm PEF. We suggested there was a threshold obtained at a certain EGCG concentration to inhibit both the cell viability and the migration, and the optimal concentration of EGCG may be altered if different parameters of PEF were applied. Hence, further studies are required to better understand the combination treatment for therapy.

In conclusion, different from a recent electrochemotherapy study that applied high intensity electric current pulse to curcumin treatment [48], we here provide a novel approach by firstly using non-invasive PEF in combination with a low concentration of herbal agent to combat against pancreatic cancer. The results of the present study indicate the combination treatment could significantly suppress the migration of cancer cells and induce apoptosis through the mitochondria-dependent pathway. For the patients, instead of having an invasive surgery and carrying out an aggressive treatment, this non-invasive combination treatment for a long-term course seemed to be more beneficial because of its mild properties to effectively inhibit cancer cells without causing severe inflammatory reactions. As a result, optimization and applications of this treatment should be continuously carried out in the future.

## Supporting information

S1 File Raw data of MTT assay. (ZIP)

S2 File Raw data of fluorescent staining (DAPI & caspase-3). (ZIP)

S3 File Raw data of Annexin V/PI flow cytometry result. (ZIP)

S4 File Raw figure of Western blot analysis (Bcl2 & Bax). (ZIP)

S5 File Raw data of DiOC_6_ flow cytometry result. (ZIP)

S6 File Raw figure of Western blot analysis (caspase-3 & -9). (ZIP)

S7 File Raw data of wound healing result. (ZIP)
