# Supplementary figures and images for: Application of non-invasive low strength pulsed electric field to EGCG treatment synergistically enhanced the inhibition effect on PANC-1 cells
Source: PLoS One. 2017 Nov 29;12(11):e0188885. doi: 10.1371/journal.pone.0188885 (PMC5706709; doi:10.1371/journal.pone.0188885)

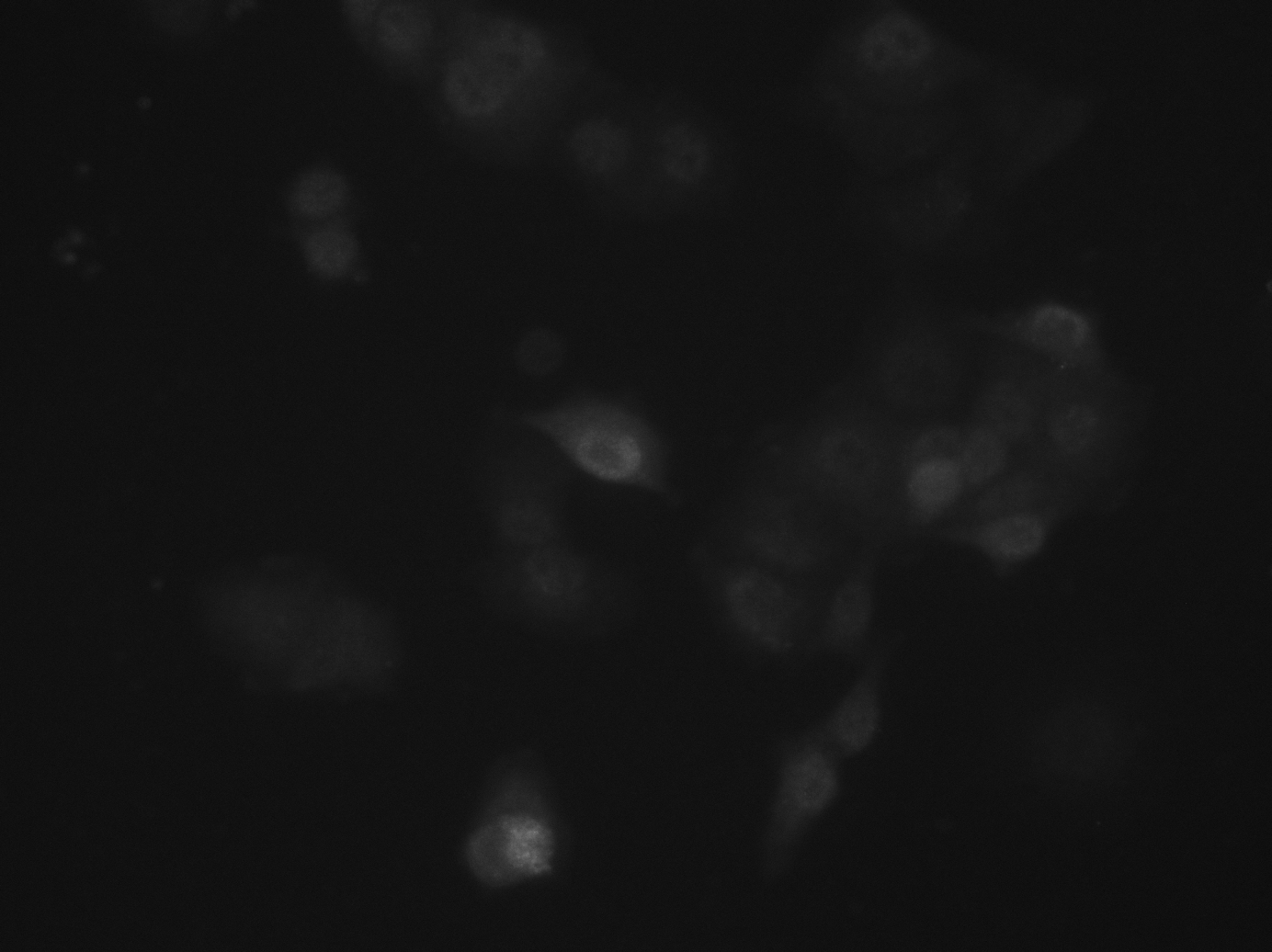

Supplement: S2 File — (ZIP) [file pone.0188885.s002.zip › DAPI+caspase3/10+PEF_CAS3.JPG]

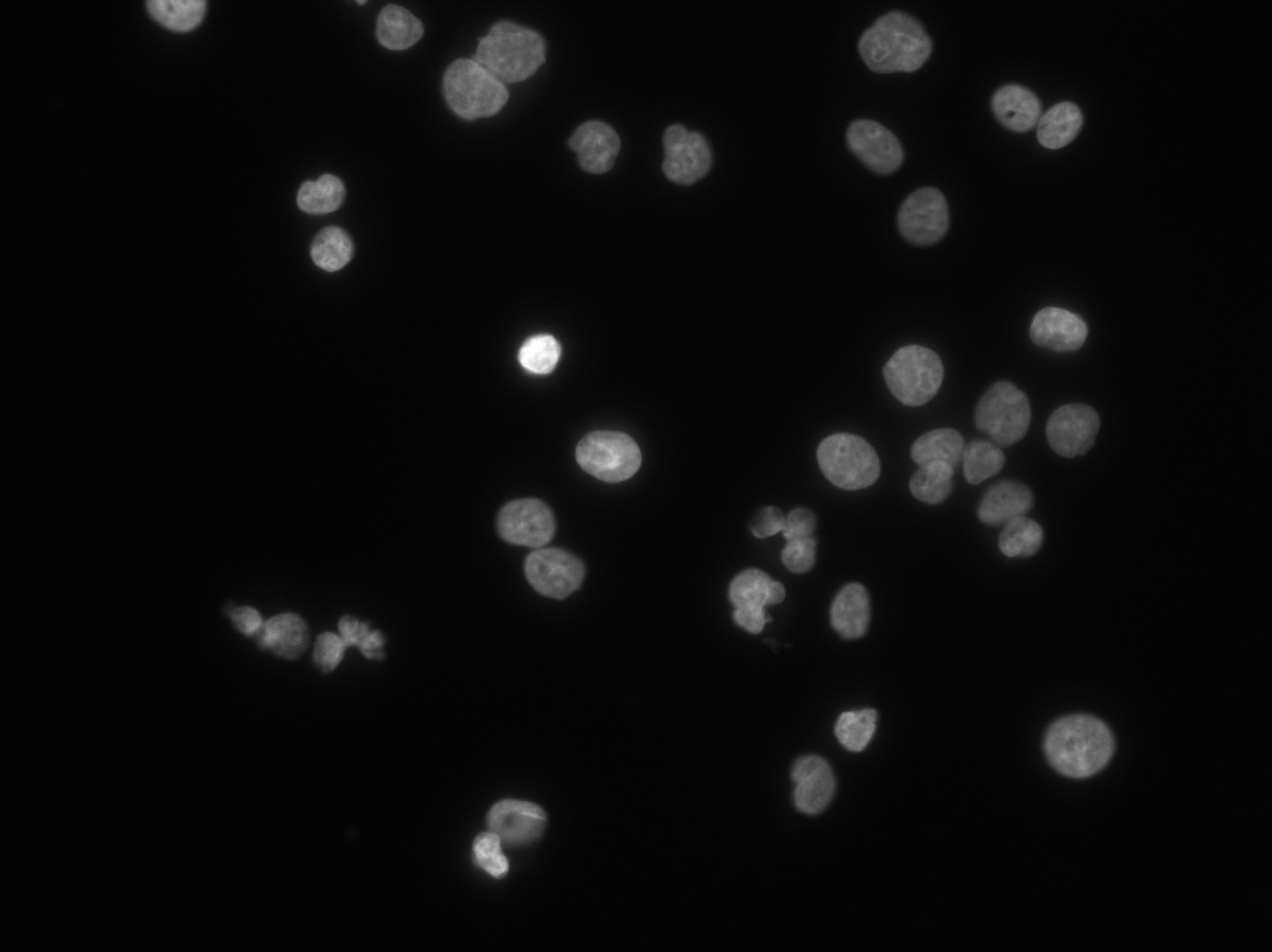

Supplement: S2 File — (ZIP) [file pone.0188885.s002.zip › DAPI+caspase3/10+PEF_DAPI.JPG]

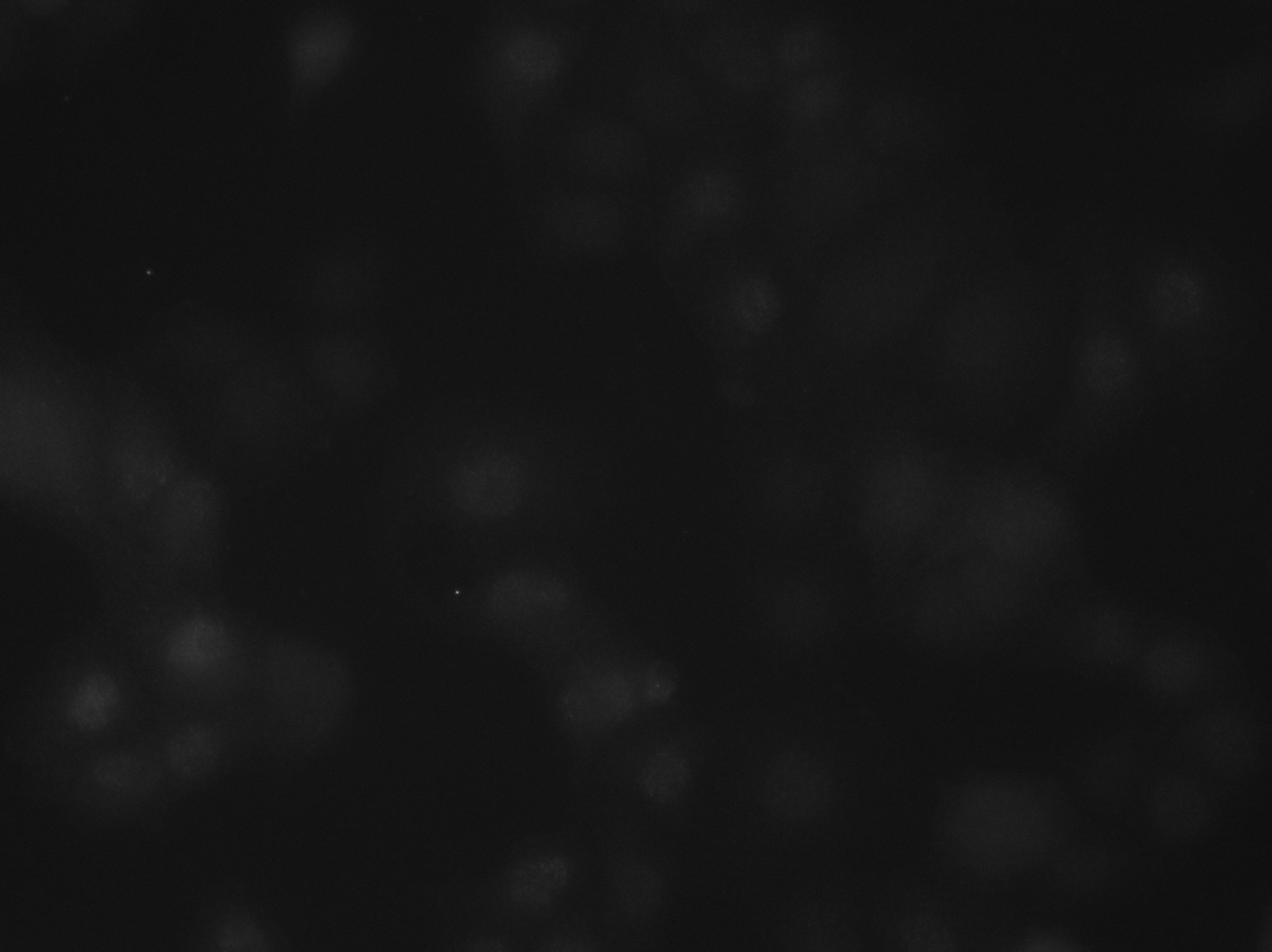

Supplement: S2 File — (ZIP) [file pone.0188885.s002.zip › DAPI+caspase3/10-40_CAS3.JPG]

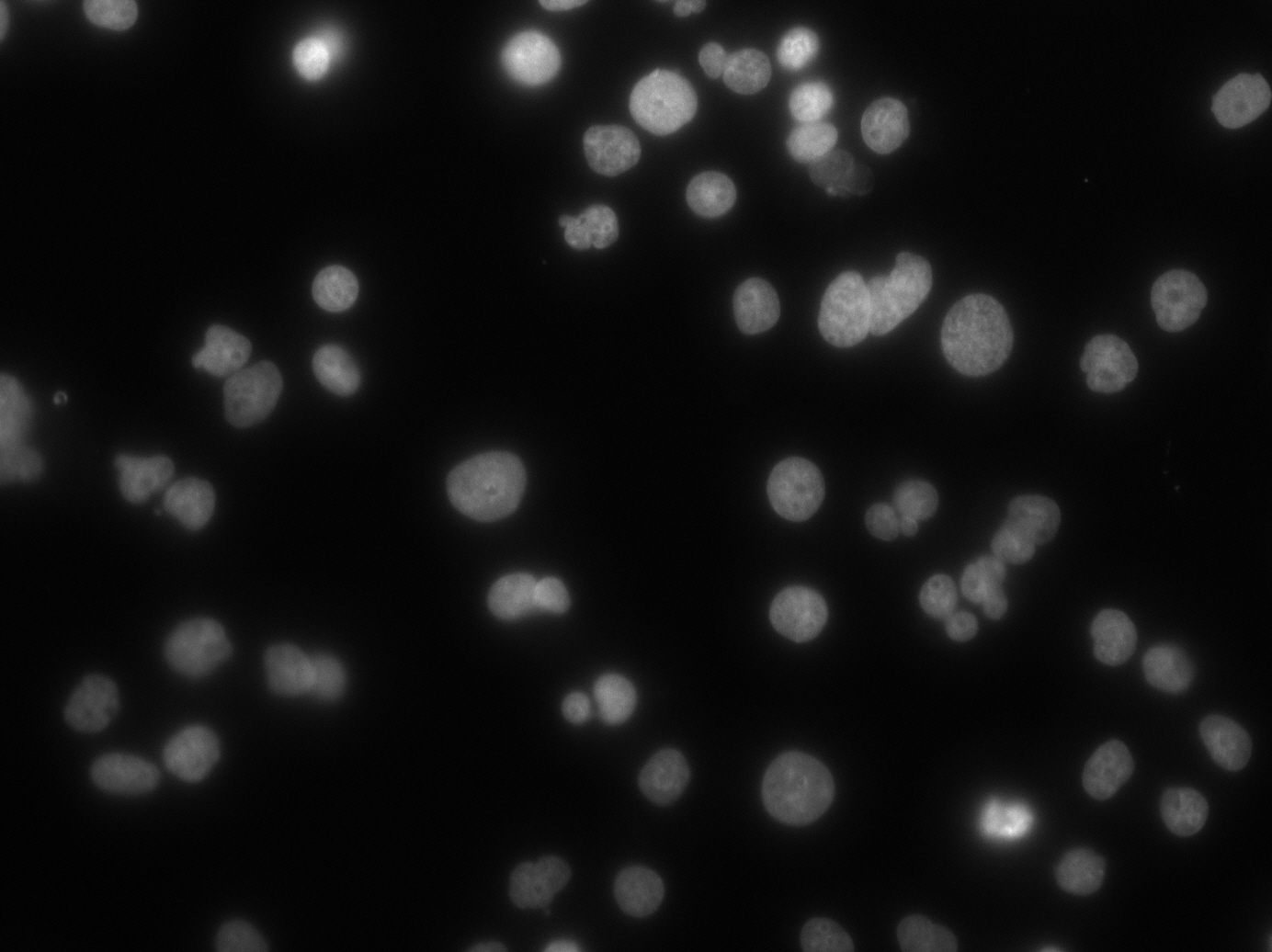

Supplement: S2 File — (ZIP) [file pone.0188885.s002.zip › DAPI+caspase3/10-40_DAPI.JPG]

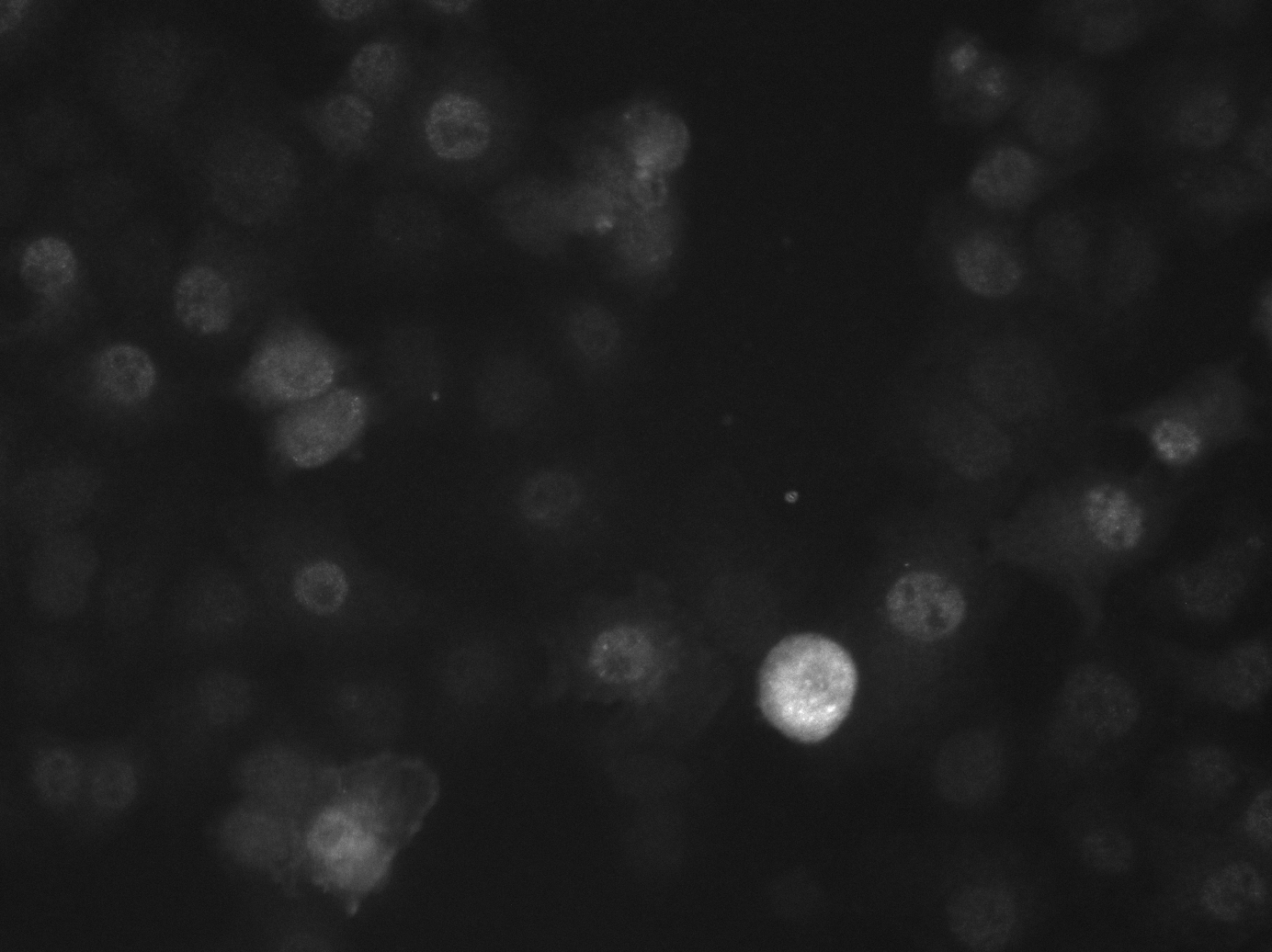

Supplement: S2 File — (ZIP) [file pone.0188885.s002.zip › DAPI+caspase3/20+PEF-40_CAS3.JPG]

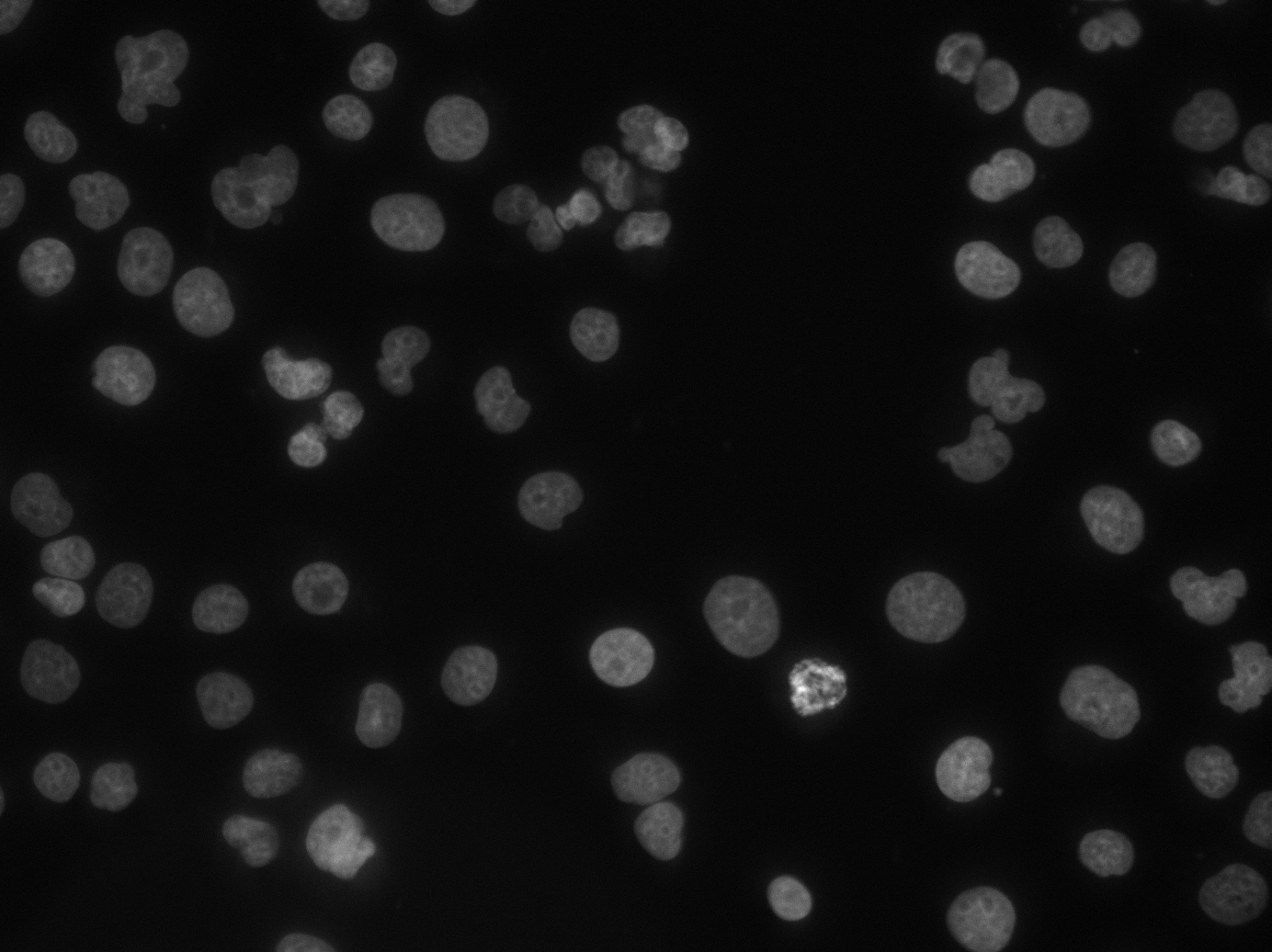

Supplement: S2 File — (ZIP) [file pone.0188885.s002.zip › DAPI+caspase3/20+PEF-40_DAPI.JPG]

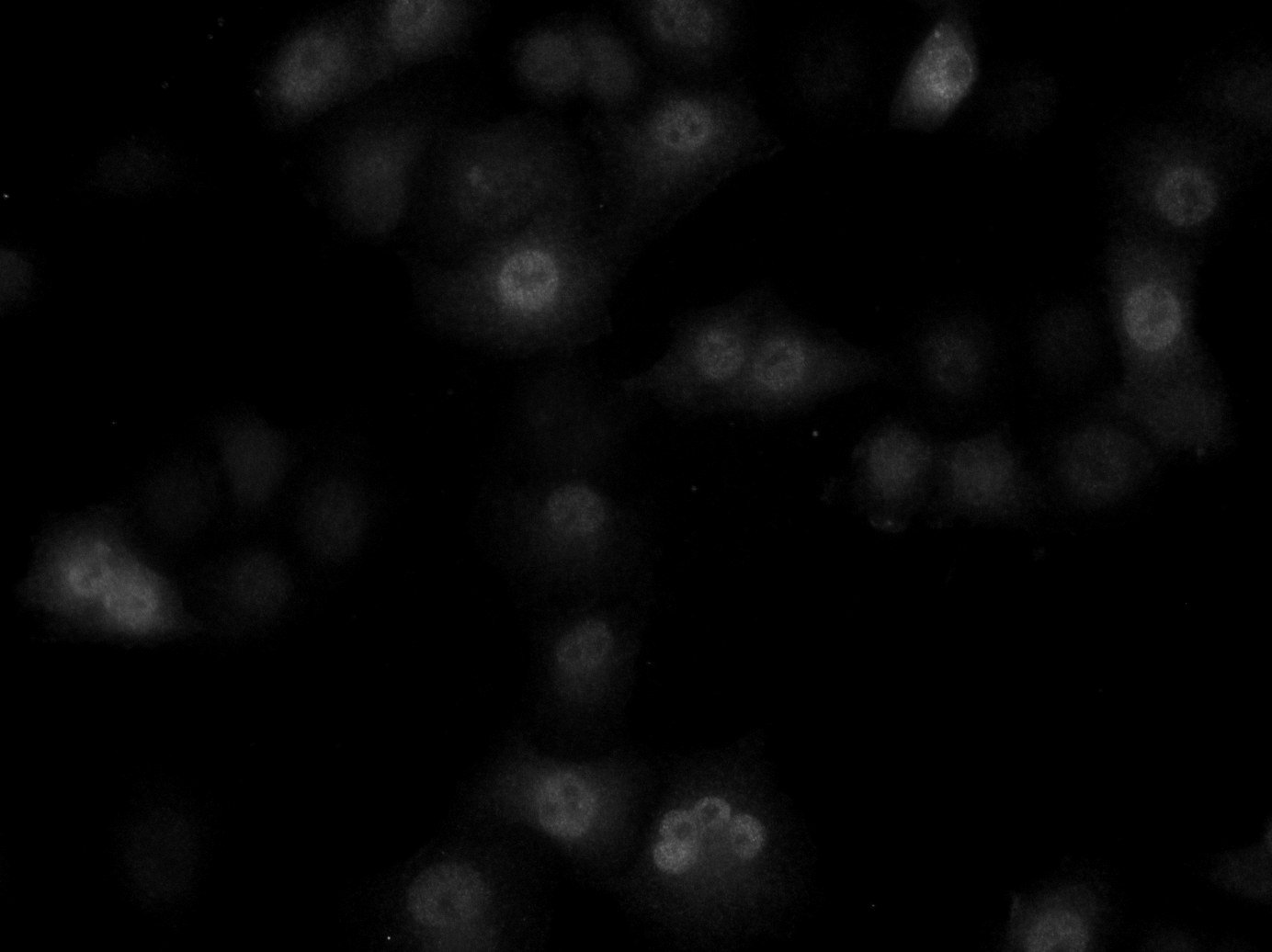

Supplement: S2 File — (ZIP) [file pone.0188885.s002.zip › DAPI+caspase3/20-40_CAS3.jpg]

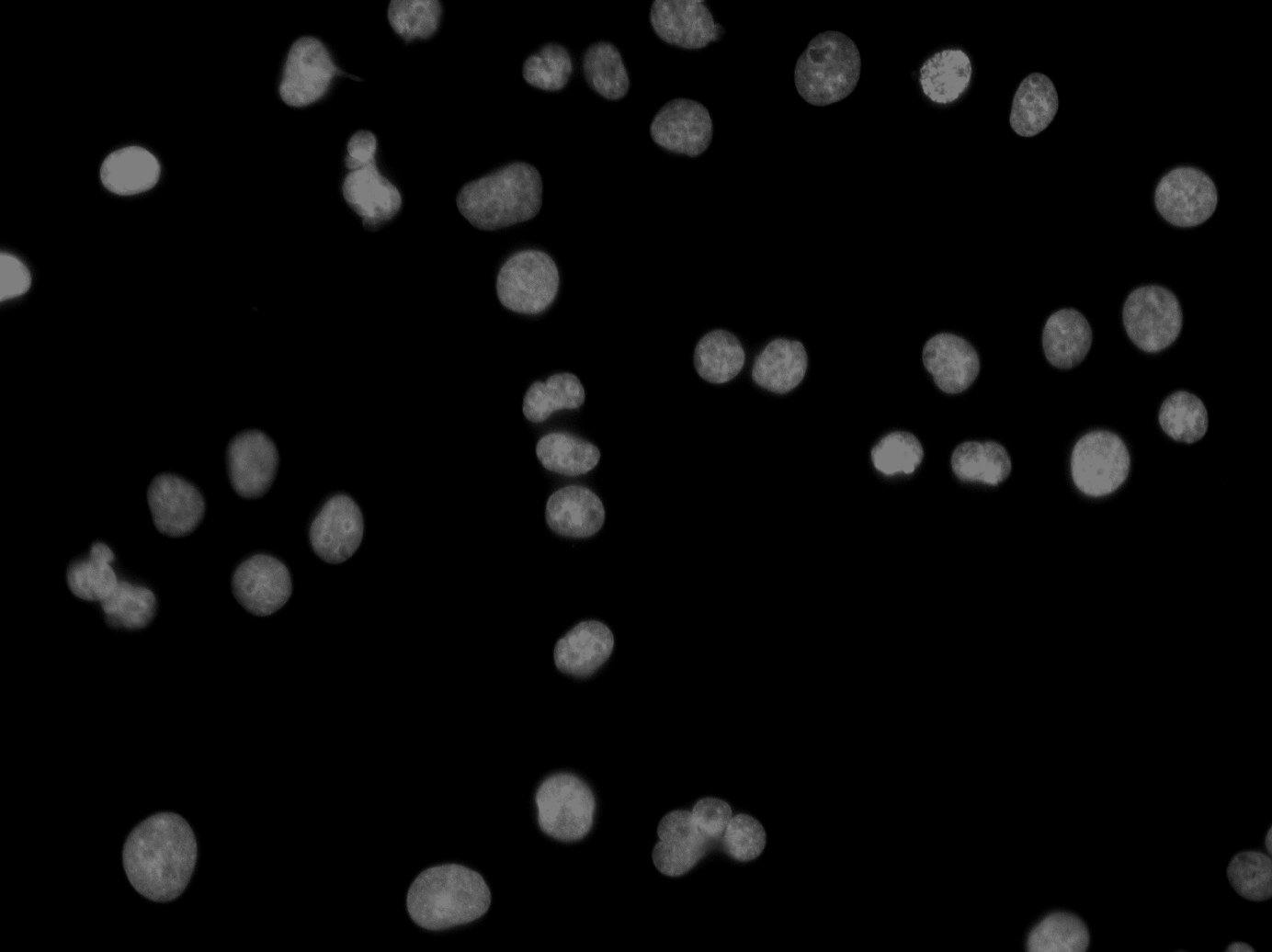

Supplement: S2 File — (ZIP) [file pone.0188885.s002.zip › DAPI+caspase3/20-40_DAPI.jpg]

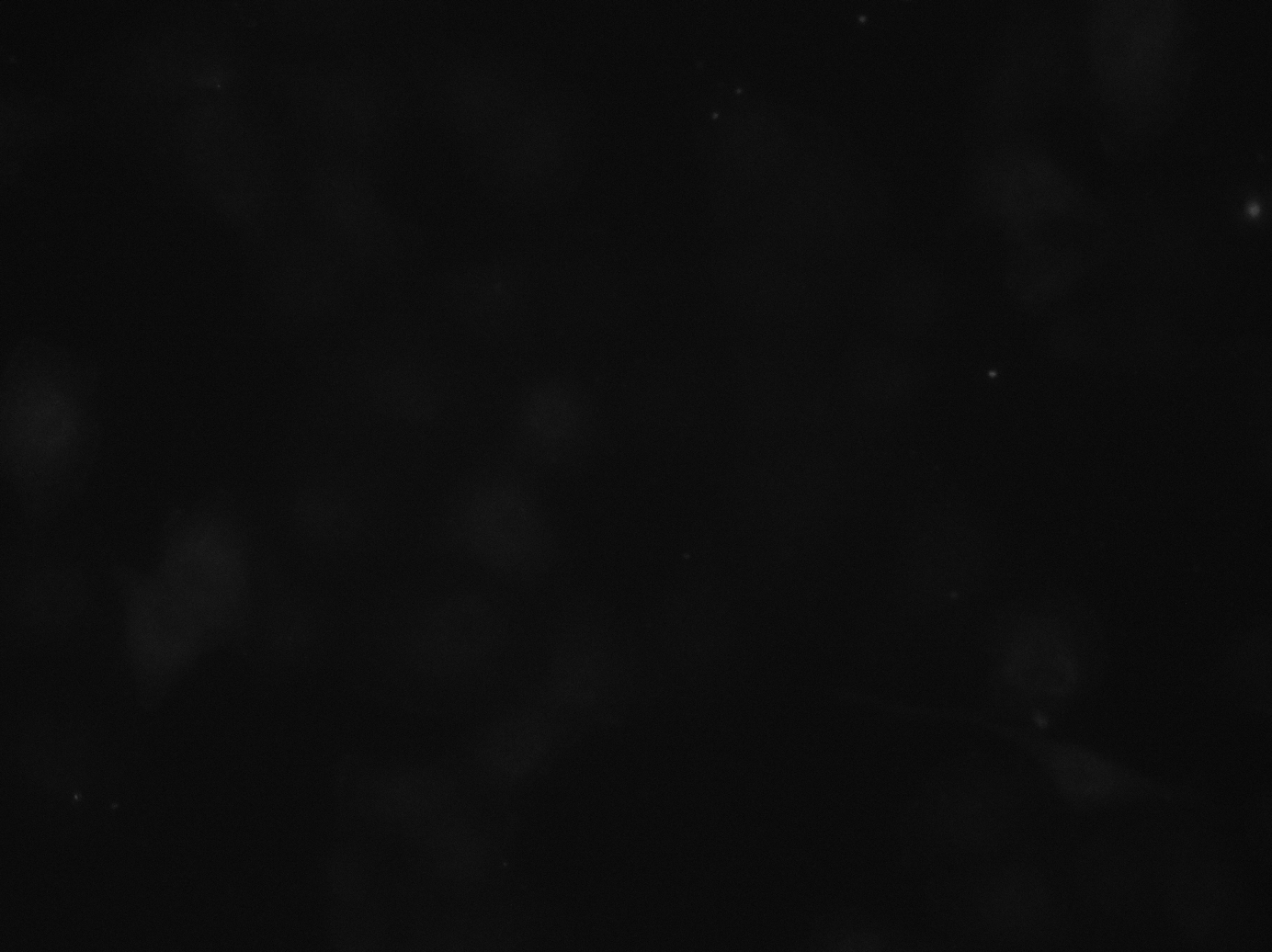

Supplement: S2 File — (ZIP) [file pone.0188885.s002.zip › DAPI+caspase3/CON-40_CAS3.JPG]

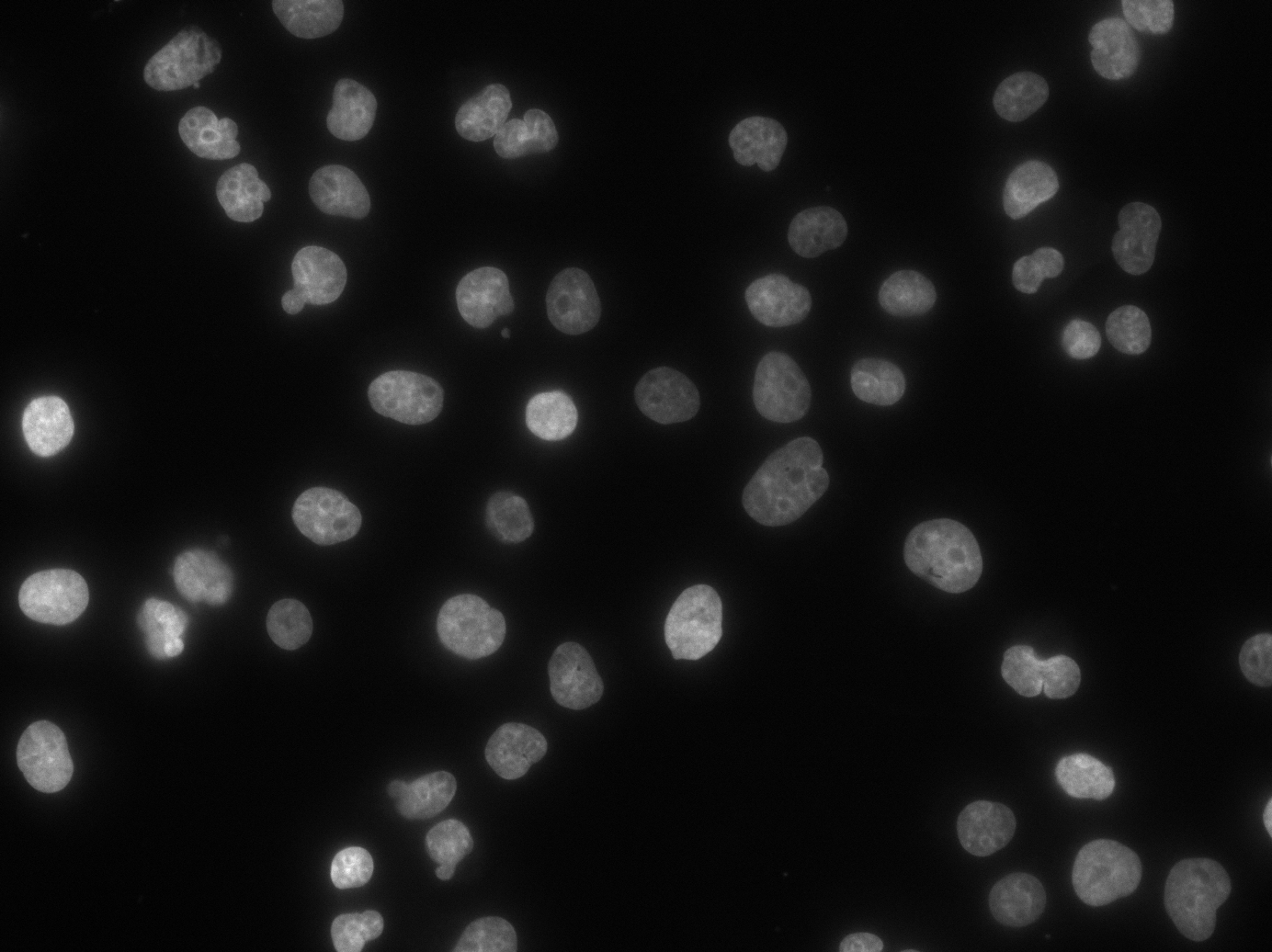

Supplement: S2 File — (ZIP) [file pone.0188885.s002.zip › DAPI+caspase3/CON-40_DAPI.JPG]

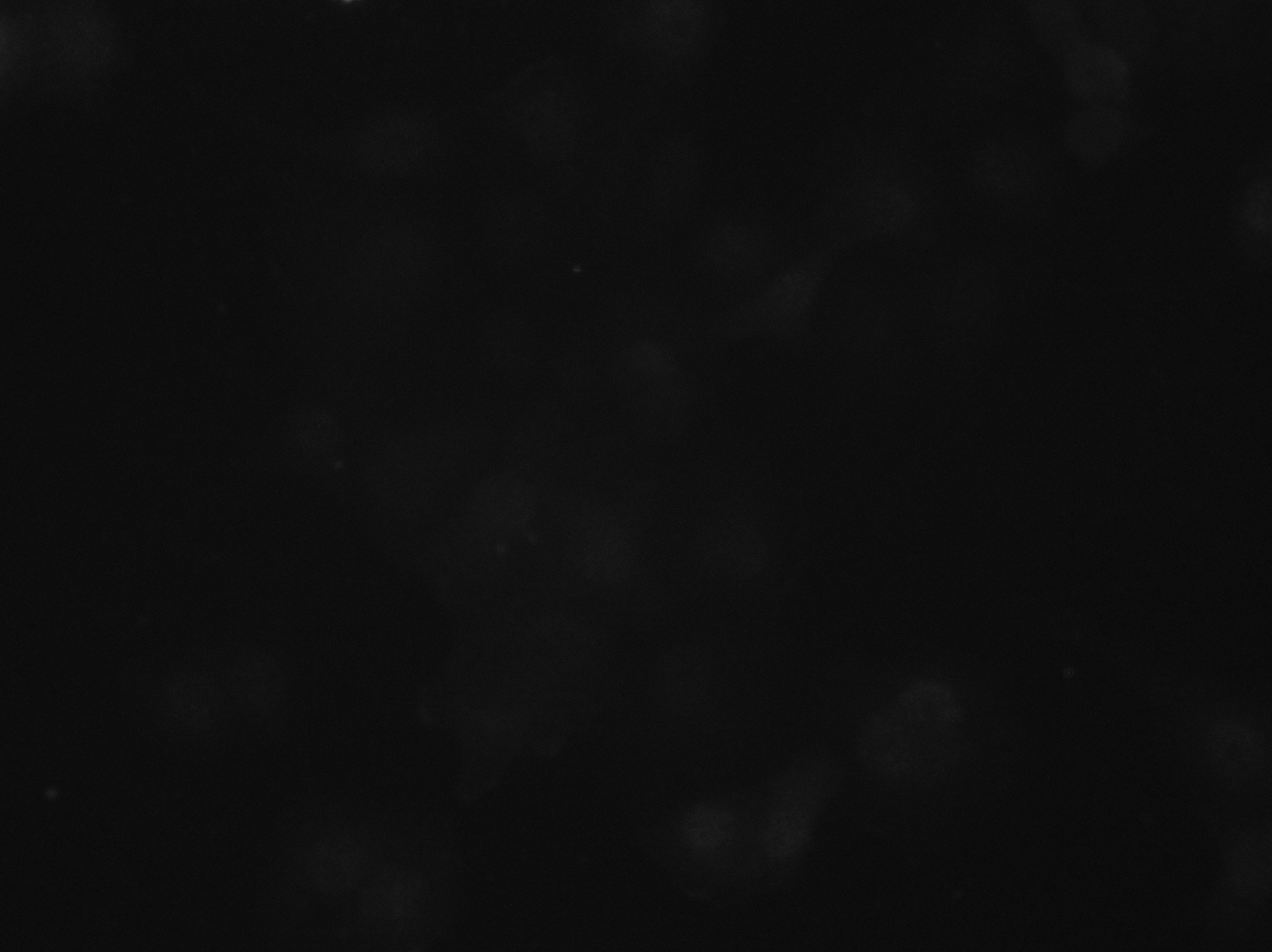

Supplement: S2 File — (ZIP) [file pone.0188885.s002.zip › DAPI+caspase3/PEF-40_CAS3.JPG]

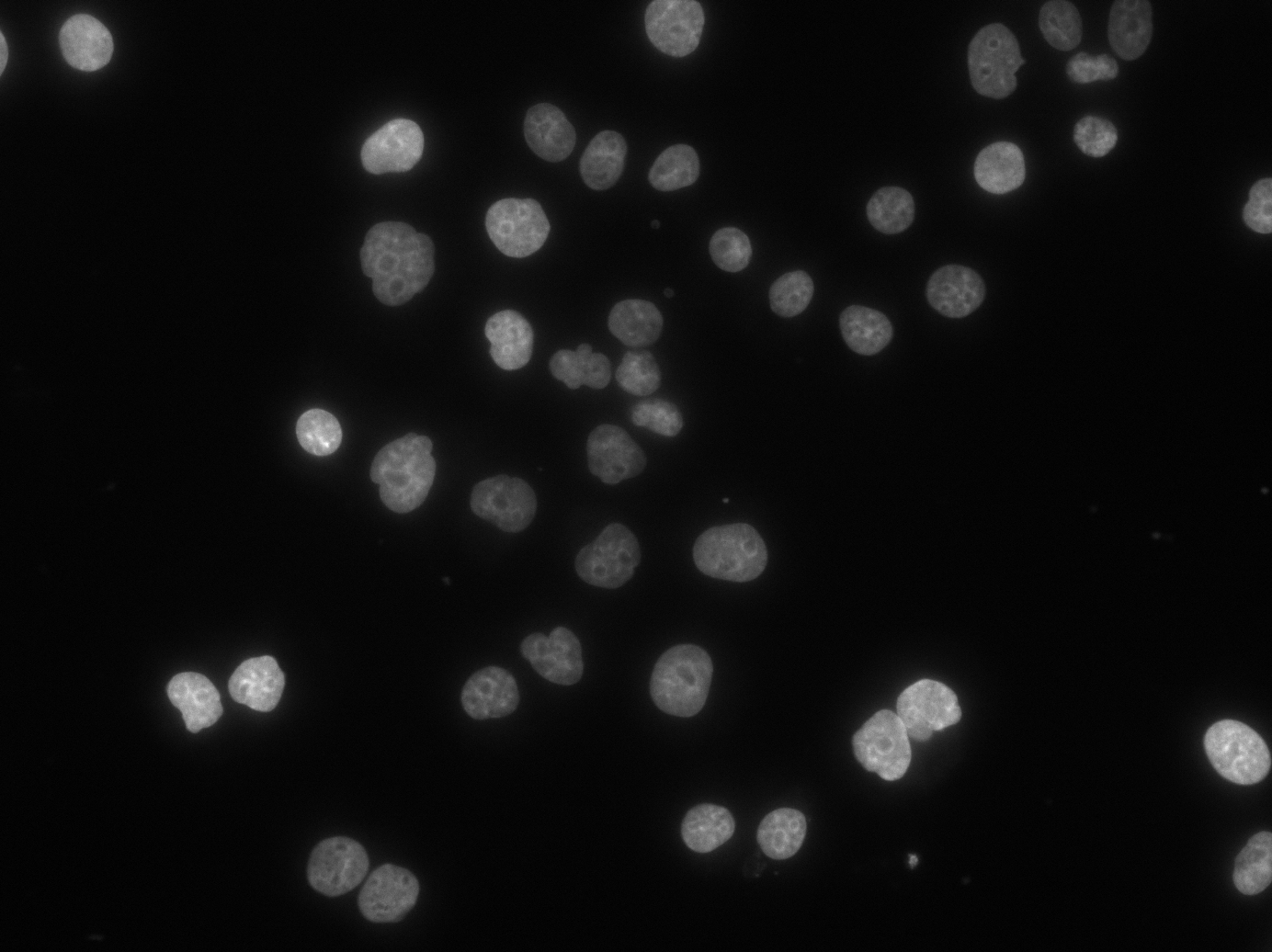

Supplement: S2 File — (ZIP) [file pone.0188885.s002.zip › DAPI+caspase3/PEF-40_DAPI.JPG]

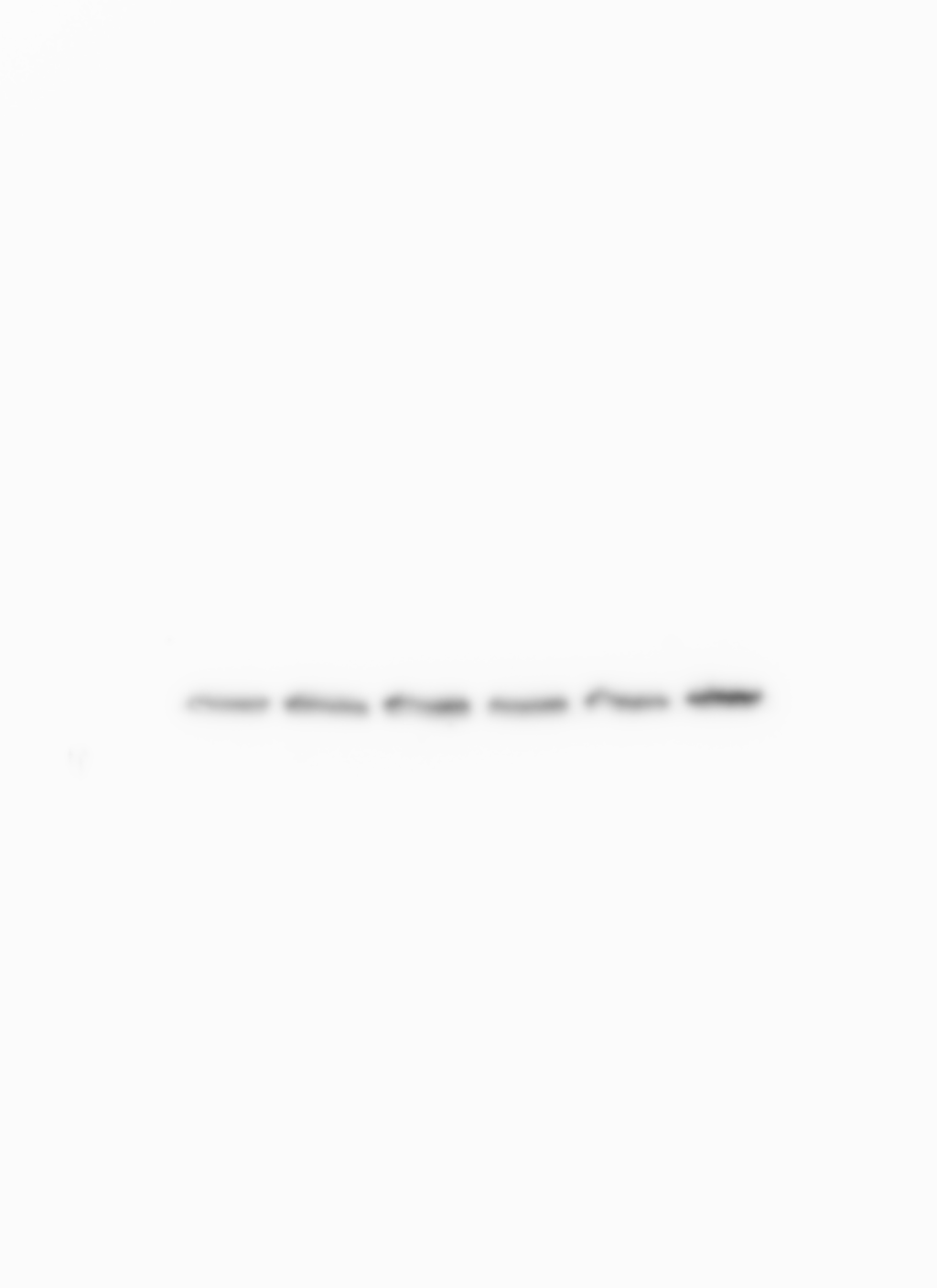

Supplement: S4 File — (ZIP) [file pone.0188885.s004.zip › bax bcl/bax bcl2/bax/Hs-P-Bax 2016.02.18_12.49.03_Ch.tif]

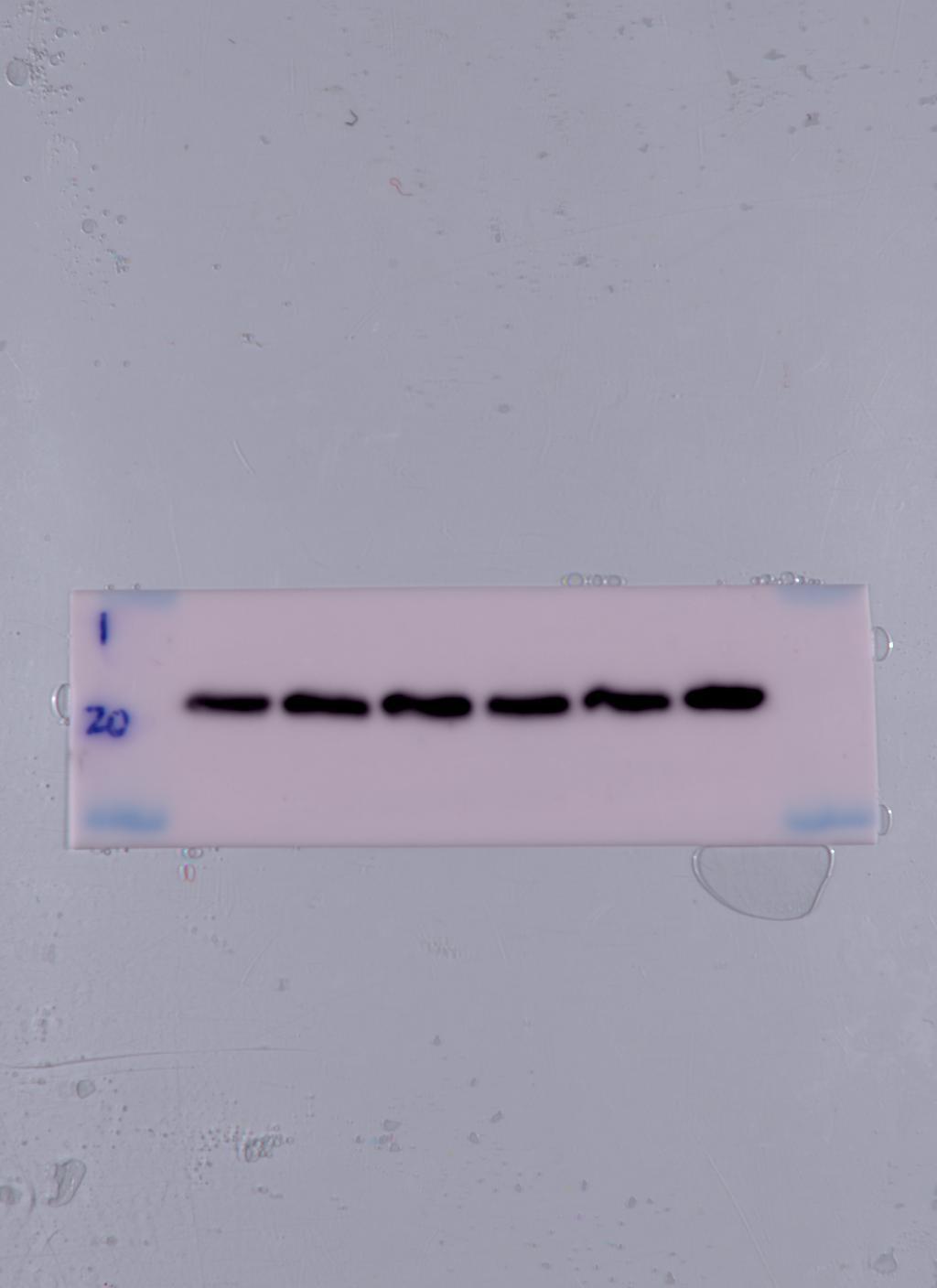

Supplement: S4 File — (ZIP) [file pone.0188885.s004.zip › bax bcl/bax bcl2/bax/Hs-P-Bax 2016.02.18_12.49.03_Ch+Marker.jpg]

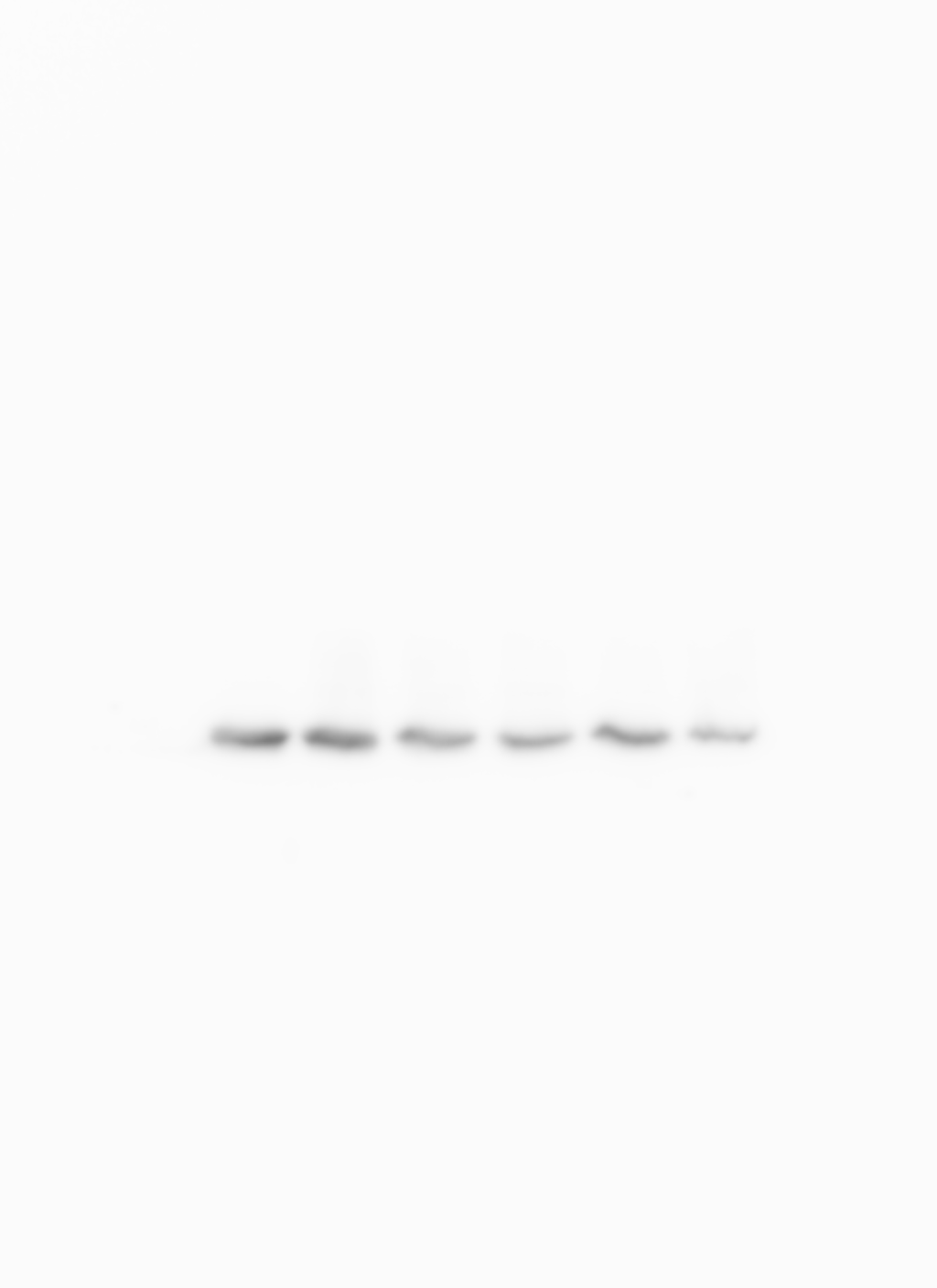

Supplement: S4 File — (ZIP) [file pone.0188885.s004.zip › bax bcl/bax bcl2/bcl/Hs-P-Bcl 2016.02.18_12.45.39_Ch.tif]

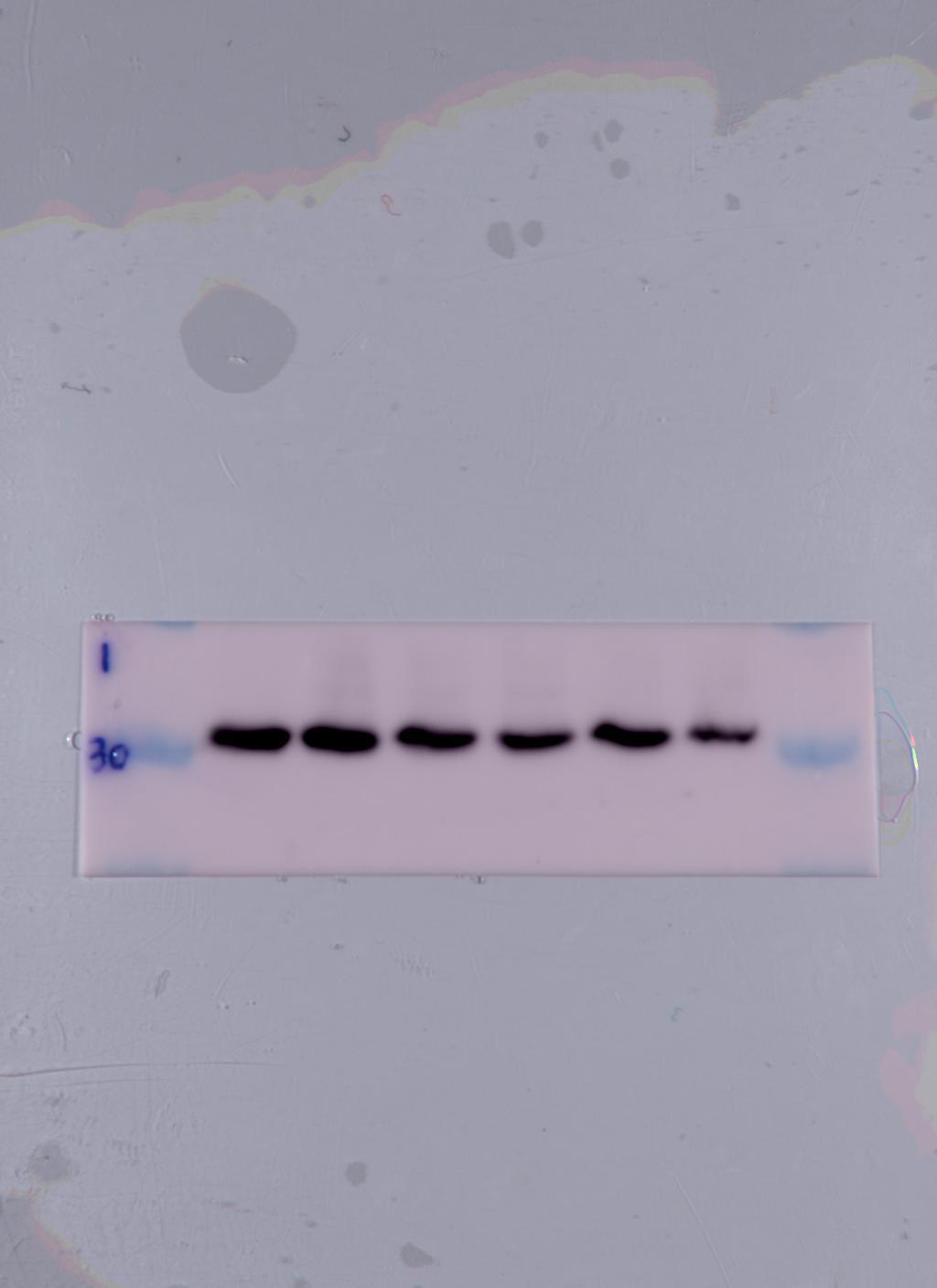

Supplement: S4 File — (ZIP) [file pone.0188885.s004.zip › bax bcl/bax bcl2/bcl/Hs-P-Bcl 2016.02.18_12.45.39_Ch+Marker.jpg]

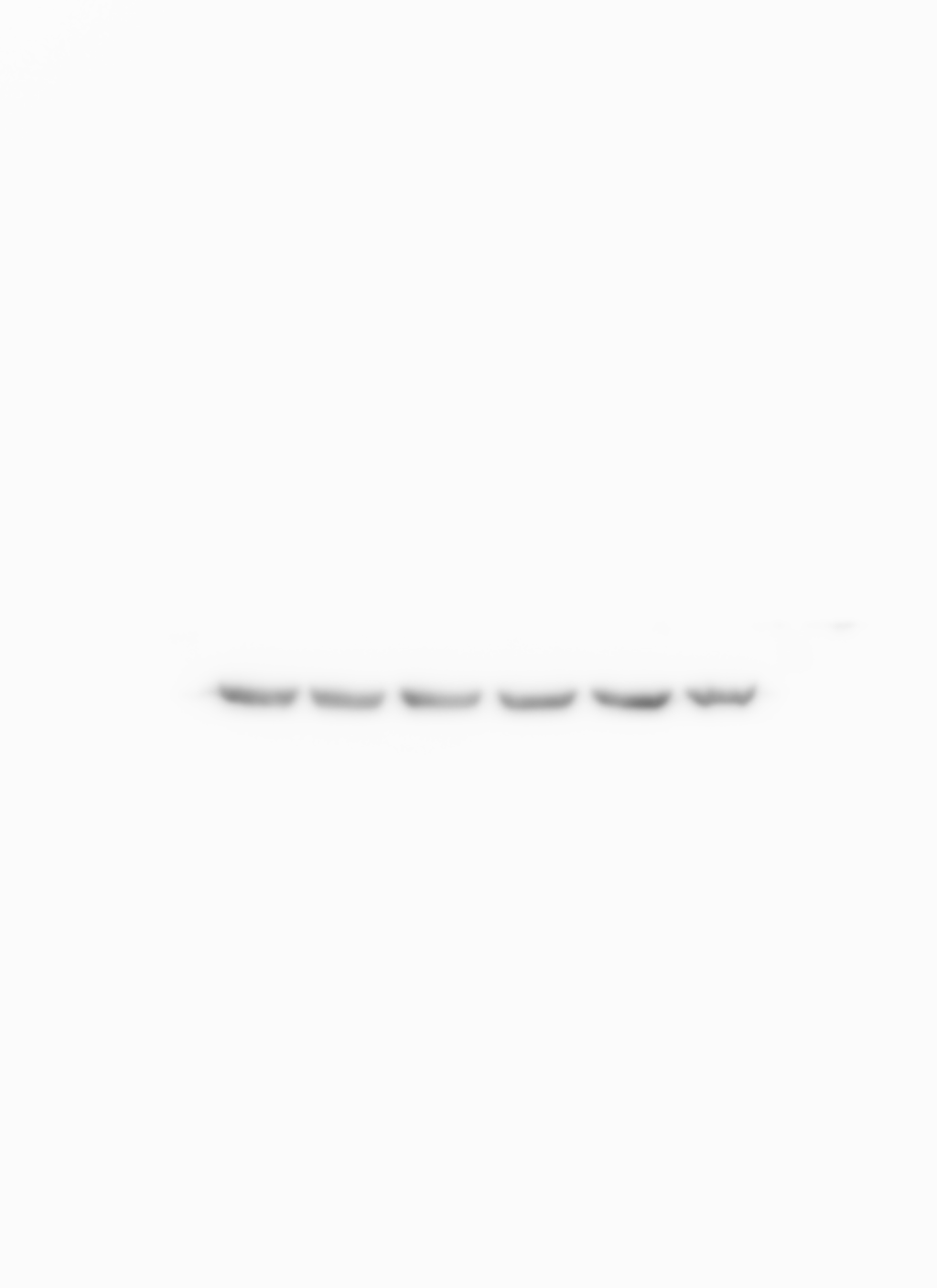

Supplement: S4 File — (ZIP) [file pone.0188885.s004.zip › bax bcl/bax bcl2/beta/Hs-P-Beta 2016.02.18_12.52.51_Ch.tif]

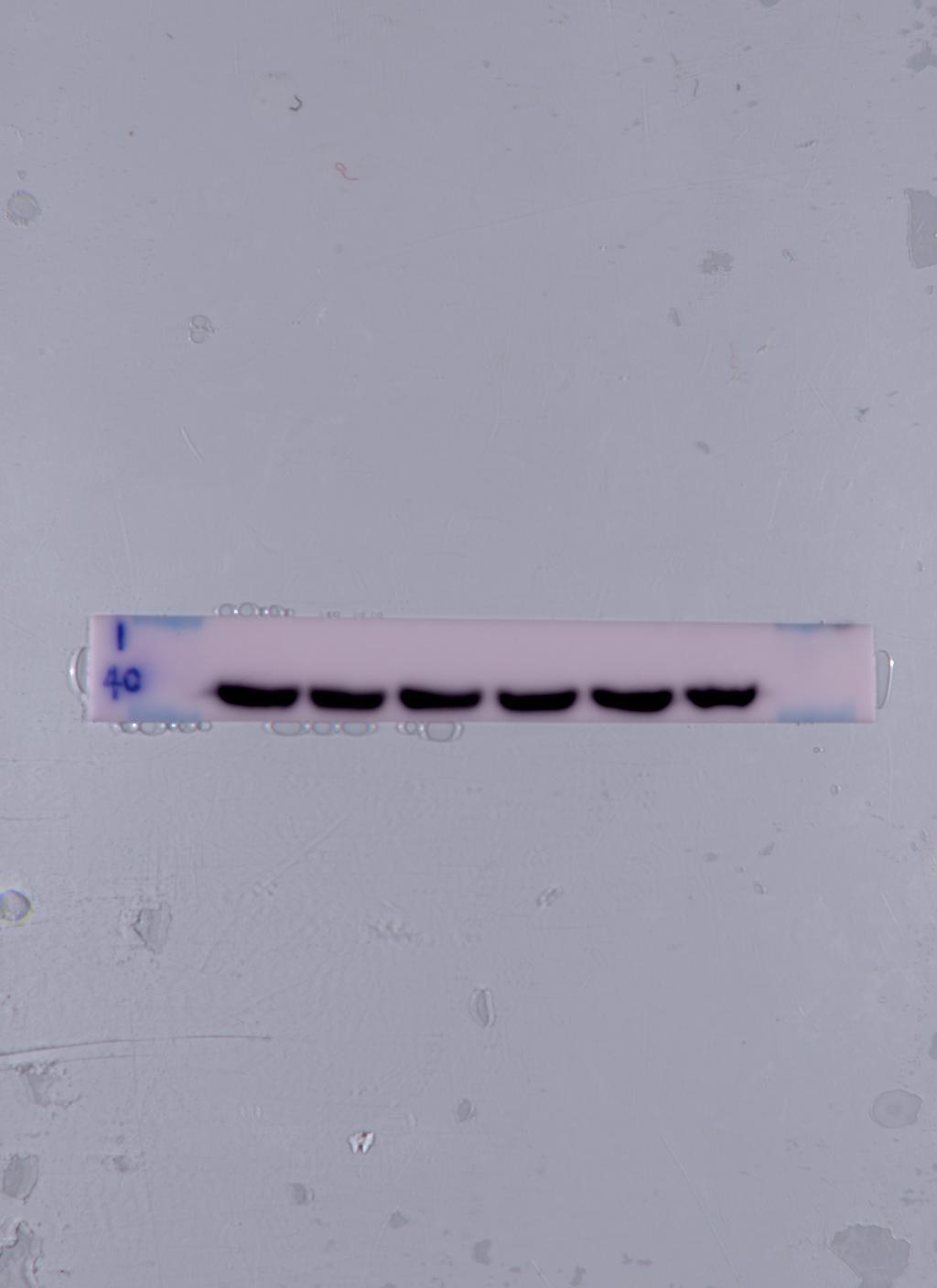

Supplement: S4 File — (ZIP) [file pone.0188885.s004.zip › bax bcl/bax bcl2/beta/Hs-P-Beta 2016.02.18_12.52.51_Ch+Marker.jpg]

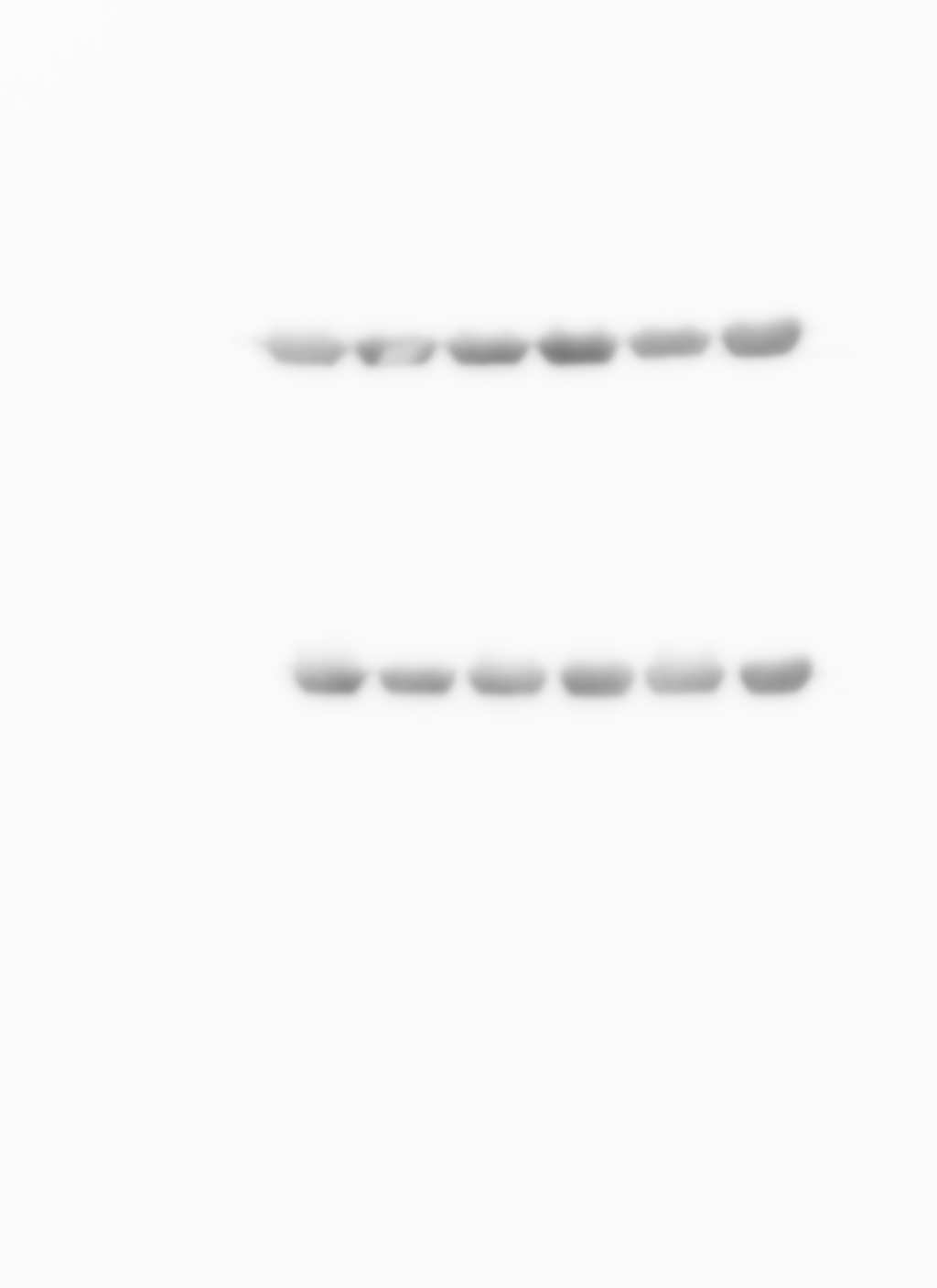

Supplement: S6 File — (ZIP) [file pone.0188885.s006.zip › caspase3 and 9/beta/Hs-P-Beta 2016.06.21_14.21.24_Ch.tif]

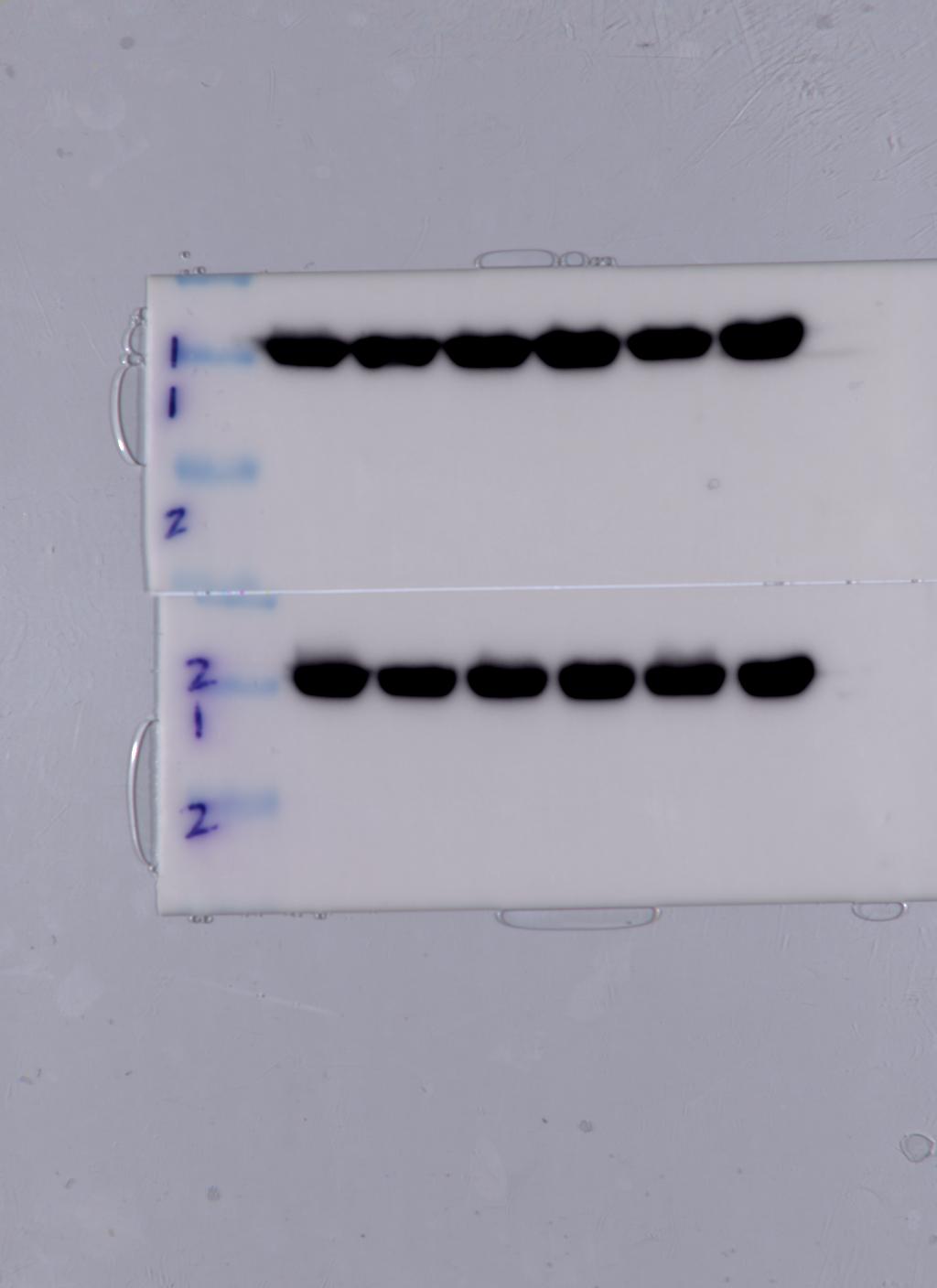

Supplement: S6 File — (ZIP) [file pone.0188885.s006.zip › caspase3 and 9/beta/Hs-P-Beta 2016.06.21_14.21.24_Ch+Marker.jpg]

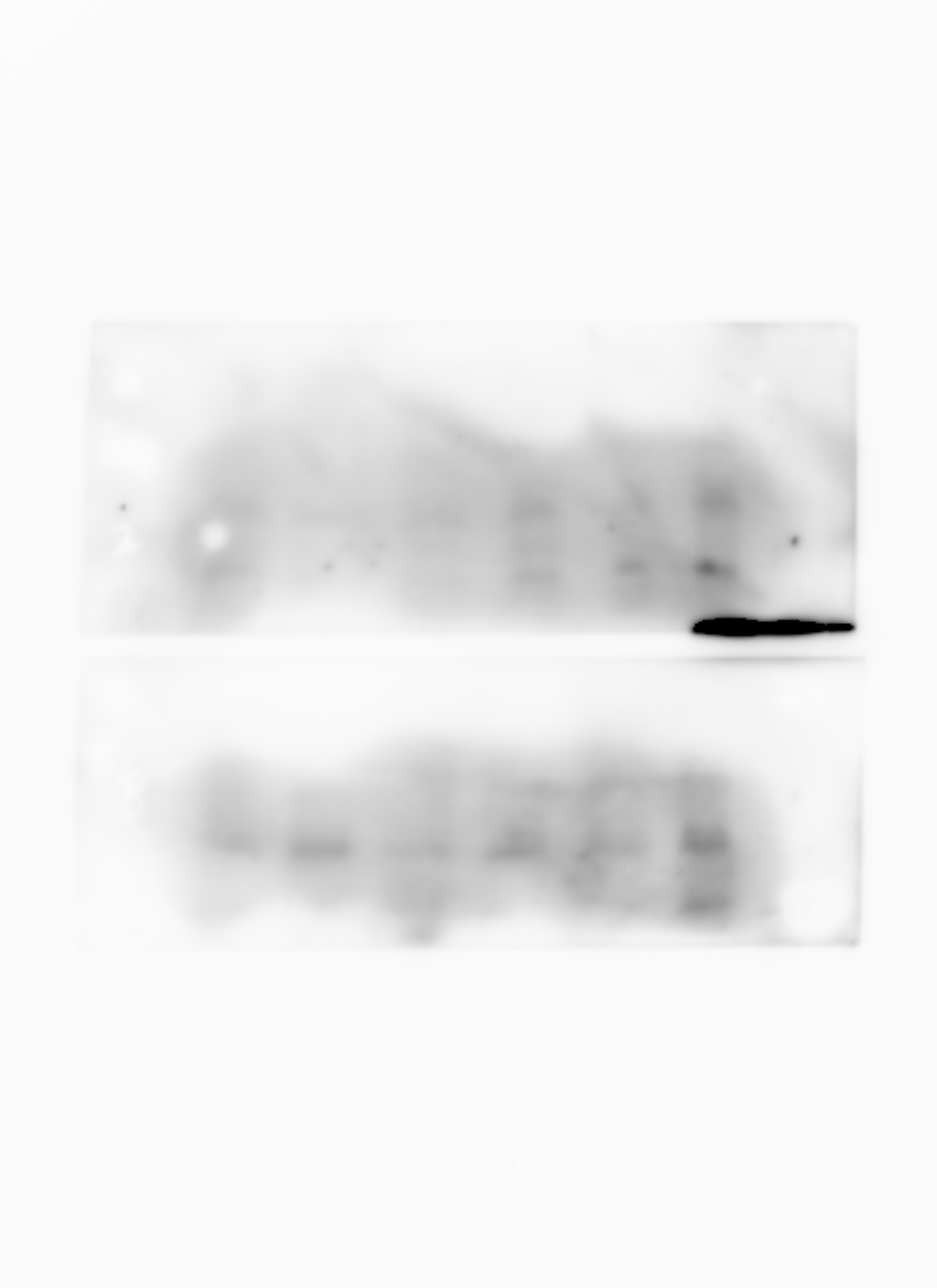

Supplement: S6 File — (ZIP) [file pone.0188885.s006.zip › caspase3 and 9/cas3/Hs-P-actcaspase3 2016.08.12_14.11.52_Ch.tif]

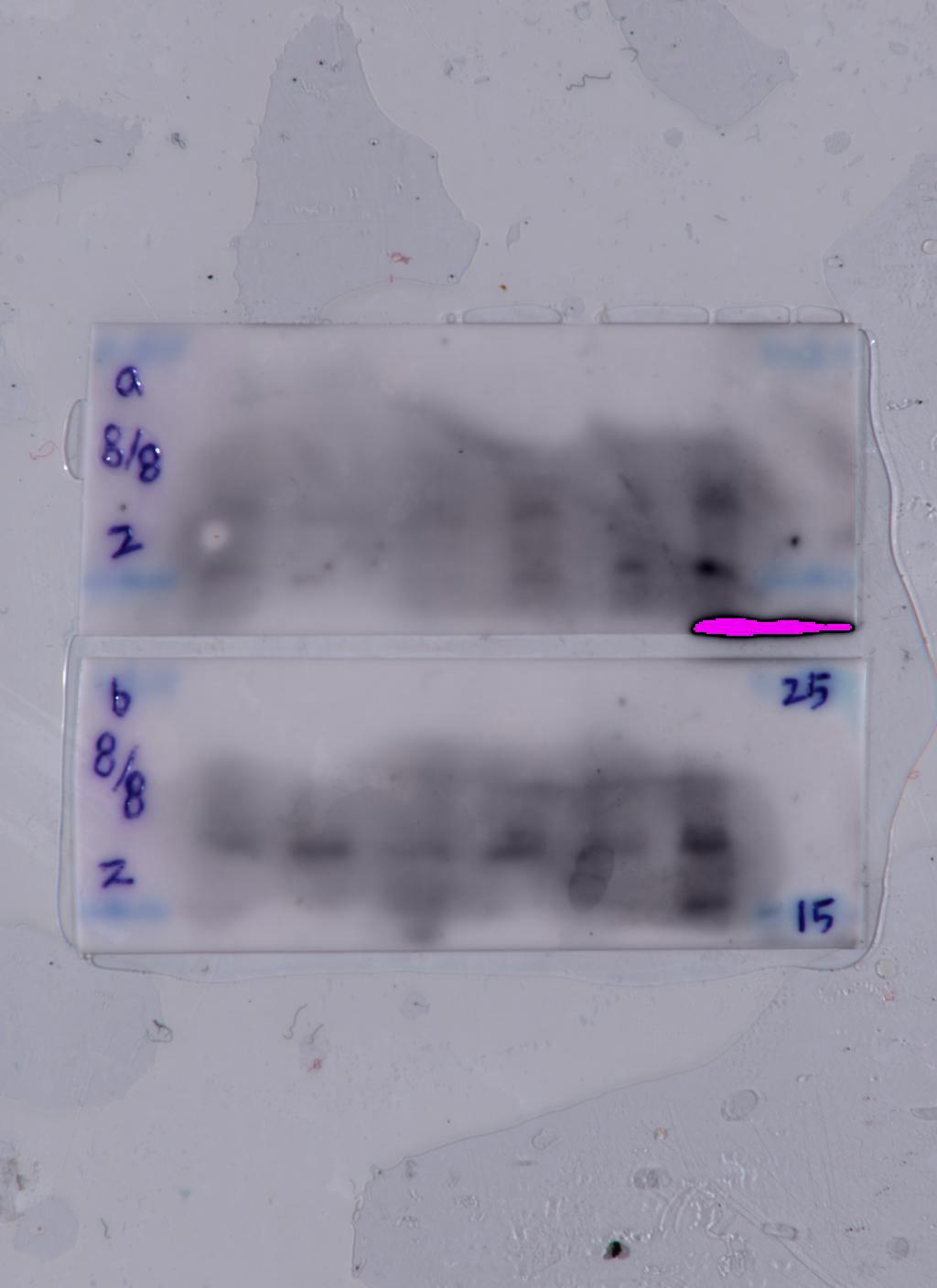

Supplement: S6 File — (ZIP) [file pone.0188885.s006.zip › caspase3 and 9/cas3/Hs-P-actcaspase3 2016.08.12_14.11.52_Ch+Marker.jpg]

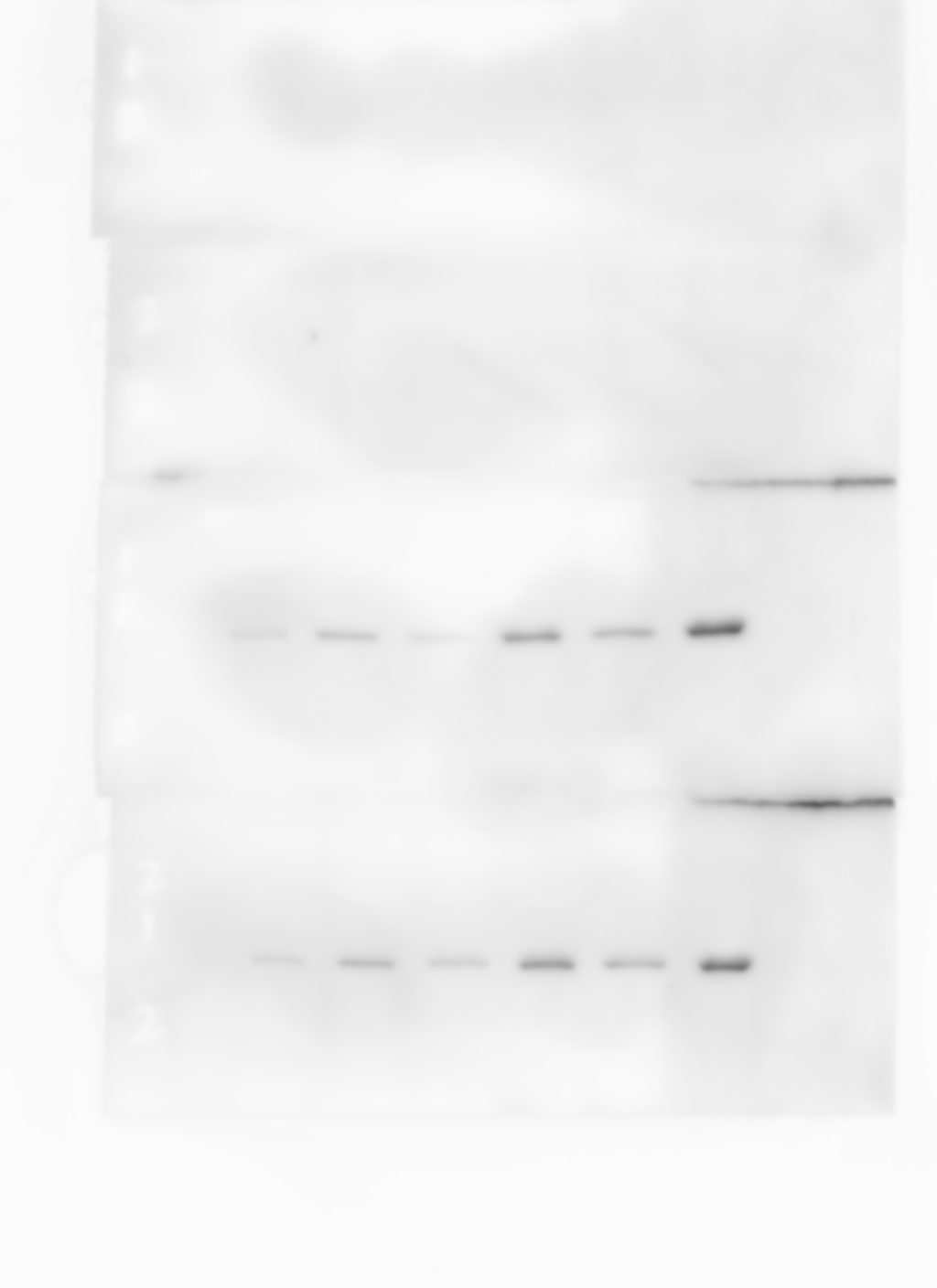

Supplement: S6 File — (ZIP) [file pone.0188885.s006.zip › caspase3 and 9/cas9/Hs-P-actcaspase9 2016.06.15_15.16.01_Ch.tif]

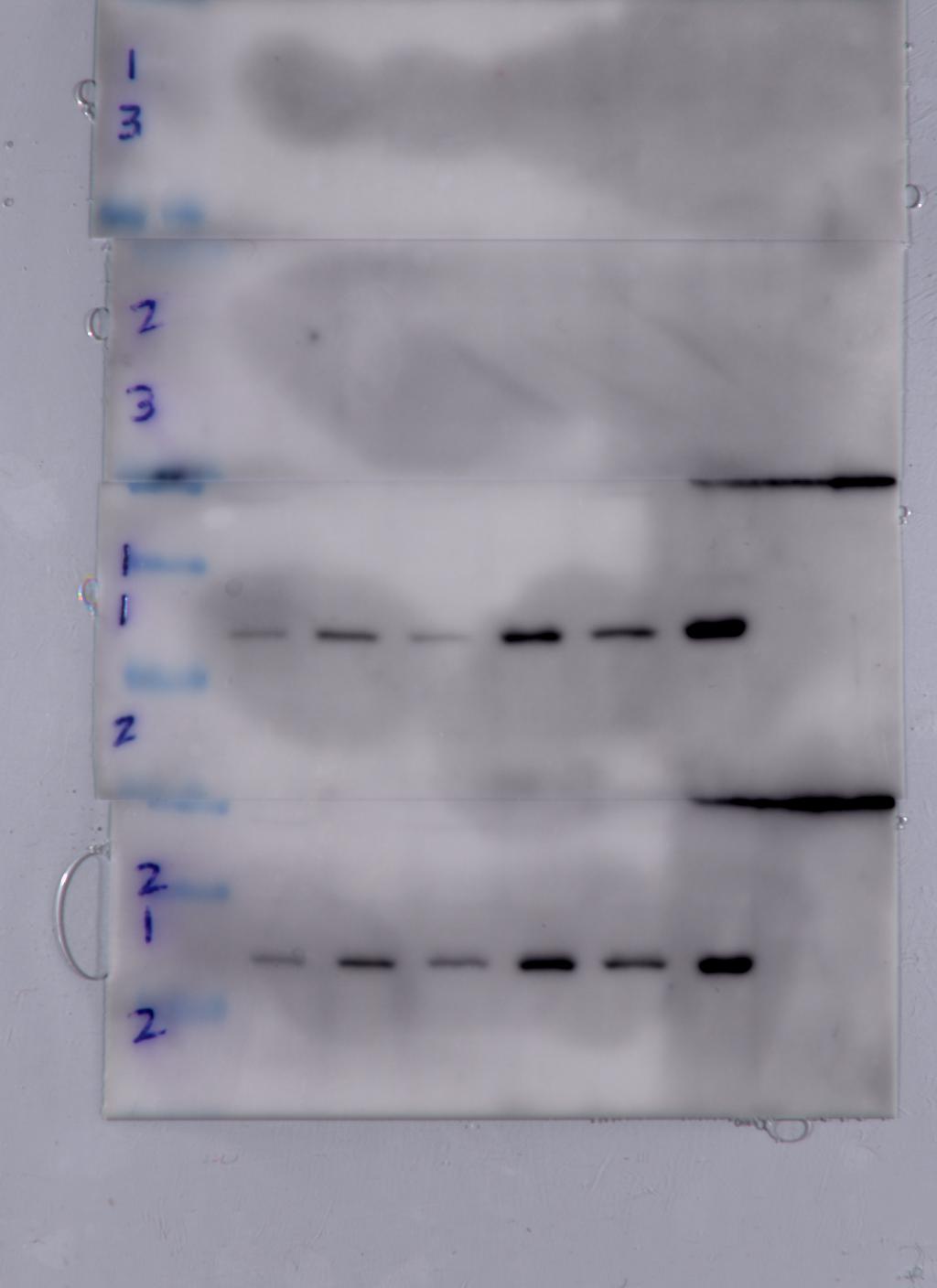

Supplement: S6 File — (ZIP) [file pone.0188885.s006.zip › caspase3 and 9/cas9/Hs-P-actcaspase9 2016.06.15_15.16.01_Ch+Marker.jpg]
